# A Plastid Protein That Evolved from Ubiquitin and Is Required for Apicoplast Protein Import in *Toxoplasma gondii*

**DOI:** 10.1128/mBio.00950-17

**Published:** 2017-06-27

**Authors:** Justin D. Fellows, Michael J. Cipriano, Swati Agrawal, Boris Striepen

**Affiliations:** a Department of Cellular Biology, University of Georgia, Athens, Georgia, USA; b Center for Tropical and Emerging Global Diseases, University of Georgia, Athens, Georgia, USA; Albert Einstein College of Medicine

**Keywords:** Toxoplasma, apicomplexan parasites, apicoplast, chloroplast, organelle protein import, ubiquitin

## Abstract

Apicomplexan parasites cause a variety of important infectious diseases, including malaria, toxoplasma encephalitis, and severe diarrhea due to *Cryptosporidium*. Most apicomplexans depend on an organelle called the apicoplast which is derived from a red algal endosymbiont. The apicoplast is essential for the parasite as the compartment of fatty acid, heme, and isoprenoid biosynthesis. The majority of the approximate 500 apicoplast proteins are nucleus encoded and have to be imported across the four membranes that surround the apicoplast. Import across the second outermost membrane of the apicoplast, the periplastid membrane, depends on an apicoplast-specific endoplasmic reticulum-associated protein degradation (ERAD) complex and on enzymes of the associated ubiquitination cascade. However, identification of an apicoplast ubiquitin associated with this machinery has long been elusive. Here we identify a plastid ubiquitin-like protein (PUBL), an apicoplast protein that is derived from a ubiquitin ancestor but that has significantly changed in its primary sequence. PUBL is distinct from known ubiquitin-like proteins, and phylogenomic analyses suggest a clade specific to apicomplexans. We demonstrate that PUBL and the AAA ATPase CDC48_AP_ both act to translocate apicoplast proteins across the periplastid membrane during protein import. Conditional null mutants and genetic complementation show that both proteins are critical for this process and for parasite survival. PUBL residues homologous to those that are required for ubiquitin conjugation onto target proteins are essential for this function, while those required for polyubiquitination and preprotein processing are dispensable. Our experiments provide a mechanistic understanding of the molecular machinery that drives protein import across the membranes of the apicoplast.

## IMPORTANCE

Apicomplexan parasites are responsible for important human diseases. There are no effective vaccines for use in humans, and drug treatment faces multiple challenges, including emerging resistance, lack of efficacy across the lifecycle, and adverse drug effects. The apicoplast is a promising target for novel treatments: this chloroplast-like organelle is derived from an algal symbiont, is absent from the host, and is essential for parasite growth and pathogenesis. We use *Toxoplasma gondii* as a model to study the apicoplast due to its strong genetic tools and established functional assays. We identify a plastid ubiquitin-like protein (PUBL) which is a novel ubiquitin-like protein and demonstrate its importance and that of the motor protein CDC48_AP_ for apicoplast protein import. These findings broaden our understanding of the evolution and mechanistic workings of a unique parasite organelle and may lead to new opportunities for treatments against important human pathogens.

## INTRODUCTION

Several important human and animal pathogens belong to the phylum *Apicomplexa*, including the parasites that cause malaria, cryptosporidiosis, and toxoplasmosis. *Toxoplasma gondii* is an intracellular parasite that infects about one-third of the human population (1). Infection usually persists throughout a person’s life, but cellular immunity restricts the parasites to chronic tissue cysts. However, loss of immune function due to various types of immunosuppression results in reactivation of the infections, which can have dire consequences for an individual’s health, including encephalitis and myocarditis (2). Another major concern is transmission of *T. gondii* from mother to fetus when initial infection of the woman occurs during pregnancy. Congenital toxoplasmosis can result in birth defects, including hydrocephalus, blindness, and stillbirths (3).

Chloroplasts are the home of photosynthesis and are the hallmark of plants and numerous multicellular algae and single-celled algal protists. Plastids evolved through endosymbiosis, a process in which a free-living photosynthetic prokaryote was taken up by a eukaryotic cell. Subsequently, different unicellular algae were engulfed by a range of eukaryotes, producing a remarkable diversity of photosynthetic organisms. Apicomplexans are among the more surprising offshoots of this evolutionary tree and have an organelle (called the apicoplast) which is derived from a secondary endosymbiotic event that took place between a red alga and a flagellated heterotrophic protist (4). While apicomplexan parasites are no longer photosynthetic, the apicoplast is essential for the parasite as the location of fatty acid, heme, and isoprenoid biosynthesis. The relative importance of each pathway differs between species and lifecycle stages and appears to be dictated by the parasite’s opportunity to scavenge host metabolites (4). The vast majority of apicoplast proteins are nucleus encoded and thus must be imported across the four membranes that surround the apicoplast in order to maintain proper organelle function (5). Nucleus-encoded apicoplast proteins are targeted to the organelle through the secretory pathway; most often, this process depends on the presence of an N-terminal bipartite leader peptide (6). The leader is made up of a signal peptide which is believed to facilitate cotranslational insertion into the endoplasmic reticulum (ER) and a transit peptide which directs proteins to the apicoplast (7). Vesicles carrying apicoplast proteins have been described in multiple reports and are thought to bud from the ER to subsequently fuse with the outermost membrane of the apicoplast (8–10). It has also been established that machinery homologous to TIC (translocon at the inner envelope membrane of chloroplasts) and TOC (translocon at the outer envelope membrane of chloroplasts), the translocons which import proteins into primary plastids, is found in the apicoplast and mediates import across the innermost and second-innermost membranes of the apicoplast, respectively (11–13). The second-outermost or periplastid membrane (PPM) is currently believed to be crossed using a specialized set of proteins that are derived from endoplasmic reticulum-associated degradation (ERAD) proteins (14). The ERAD machinery typically acts as a quality control system for protein folding in the ER and the secretory pathway. Misfolded proteins are recognized and exported across the ER membrane, where they are marked by ubiquitination, leading to subsequent degradation by the proteasome (15).

We have previously provided genetic evidence for a model in which the apicoplast ERAD machinery, including the ubiquitin (UB) conjugating enzyme (E2_AP_), has been retooled for protein import rather than protein degradation (16). While multiple ERAD components have been identified in the apicoplast of *T. gondii*, including CDC48_AP_ and Ufd1_AP_, their function and potential interaction with the ubiquitin machinery are still unclear (14). In the ERAD system, CDC48 is a hexameric AAA ATPase that provides the mechanical force to unfold and extract misfolded proteins across the ER membrane (17). It is presently unclear how apicoplast-imported proteins are recognized by the import machinery, as the apicoplast ERAD machinery is reduced compared to that of other complex plastids, which contain additional plastid ERAD components (18). We propose that CDC48_AP_ and the ubiquitination machinery act in recognizing proteins at the PPM and in transporting apicoplast proteins across the membrane. This idea is supported by the findings that CDC48 and its cofactor Ufd1 have ubiquitin binding domains and that ubiquitin recognition is necessary for proper translocation of misfolded proteins across the ER membrane (19, 20). Similarly, study results suggest that ubiquitination in the ERAD system has an initial mechanistic role in protein translocation across the ER membrane in addition to its subsequent role in protein degradation (21). In addition, the ER membrane spanning ubiquitin ligase is key in substrate recognition and its autoubiquitination is mandatory for translocation of substrates (22, 23).

In this report, we identify a ubiquitin-like protein that is localized to the apicoplast and differs in its amino acid sequence significantly from known ubiquitin-like proteins. We provide genetic evidence that the ubiquitin-like protein and CDC48_AP_ are critical for parasite survival and import across the PPM of the apicoplast. We also demonstrate that the C-terminal diglycine motif of this ubiquitin-like protein is critical to its function. The data suggest that conjugation of the ubiquitin-like protein onto imported proteins and the ATPase domain of CDC48_AP_ is a mechanistic requirement for import into the apicoplast.

## RESULTS

### CDC48_AP_ is critical for parasite survival and protein import into the apicoplast.

**FIG 1:**
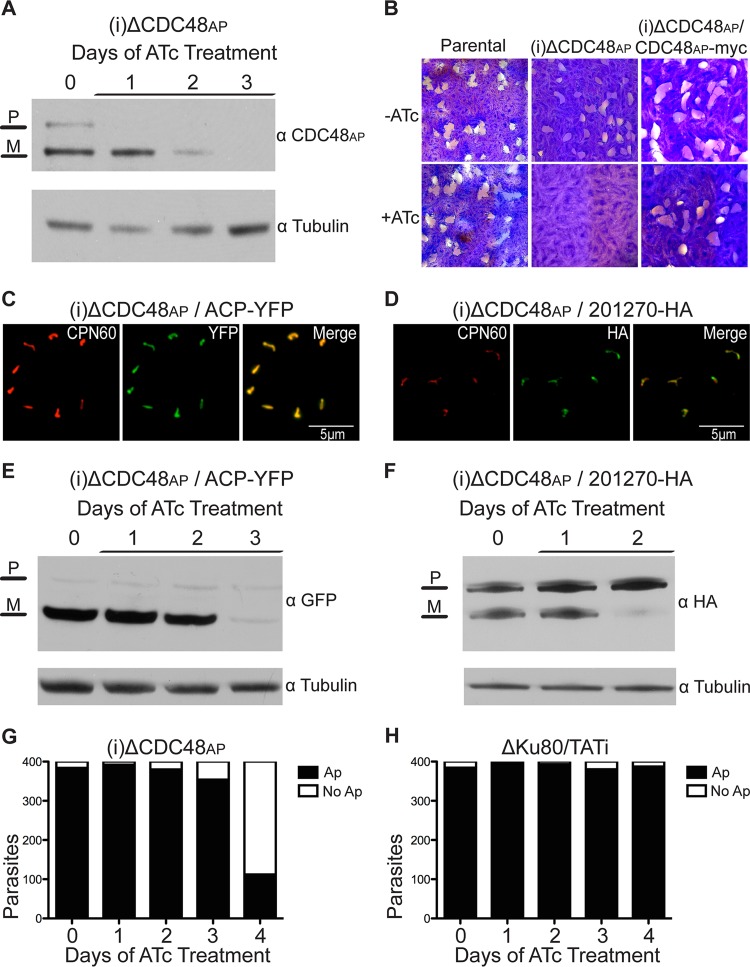
CDC48_AP_ is critical for parasite survival and import across the PPM. (A) Western blot analysis using CDC48_AP_ antibody and the (i)ΔCDC48_AP_ line after ATc treatment for the indicated times. Levels of CDC48_AP_ were diminished after 1 day of ATc treatment and were completely ablated after 3 days of ATc treatment. P, precursor; M, mature band. (B) Plaque assays were performed on the parental ΔKu80/TATi line, the (i)ΔCDC48_AP_ line, and the complemented (i)ΔCDC48_AP_ line in the absence or presence of 0.5 μg/ml of ATc. Note the lack of plaque formation for (i)ΔCDC48_AP_ under conditions of ATc treatment. (C and D) Immunofluorescence assays performed on stable (i)ΔCDC48_AP_ lines expressing ACP-YFP (C) or 201270-HA (D). CPN60 (red) served as a marker for the apicoplast. ACP-YFP and 201270-HA (both green) are properly localized to the apicoplast and show patterns of overlap of CPN60 typical for *T. gondii* luminal or PPC apicoplast proteins. (E and F) Apicoplast import assays were performed with the (i)ΔCDC48_AP_/ACP-YFP line (E) and the (i)ΔCDC48_AP_/201270-HA line (F). Parasites were treated with ATc for the time indicated and harvested for Western blot analysis using YFP antibody and HA antibody, respectively. Note the loss of the mature band (M) for both reporters, while the levels of the precursor band (P) remained unchanged under ATc treatment. (G) The presence of apicoplasts (Ap) was scored daily by IFA using anti-CPN60 for 400 ATc-treated (i)ΔCDC48_AP_ parasites. (H) The same assay was performed on the parental ΔKu80/TATi lines. Note that there was no significant difference in apicoplast numbers after ATc treatment. Anti-tubulin served as a loading control in the experiments whose results are shown in panels A, E, and F.

CDC48 is a highly conserved protein found in a wide array of eukaryotic organisms. CDC48 is an AAA ATPase typically localized to the cytoplasm, where it is involved in a multitude of functions, including cell cycle regulation, transcriptional activation, apoptosis, autophagy, endolysosomal sorting, and ER-associated degradation (Erad) (24). In these different contexts, CDC48 uses the energy of ATP hydrolysis to unfold proteins, disassemble protein complexes, or translocate proteins across membranes. We have previously shown that there are two distinct CDC48 proteins in *T. gondii*; one is localized to the cytoplasm whereas the other (CDC48_AP_) is localized to the periplastid compartment (PPC) of the apicoplast (14). This is consistent with the idea that CDC48_AP_ is part of the ERAD-derived complex that aids proteins in crossing the periplastid membrane (PPM) of the apicoplast. Specifically, we hypothesize that CDC48_AP_ acts as the motor of the translocon. However, our initial attempts to test this and to generate a conditional mutant using a regulated ectopic copy and targeting plasmids failed. We thus modified a fosmid containing the CDC48_AP_ locus to replace its promoter (25). The engineered fosmid was transfected into a parasite line that is limited to homologous recombination and carries a tetracycline (Tc)-repressible transactivator (ΔKu80/TATi) and was selected in the presence of pyrimethamine to isolate a stable line carrying a CDC48_AP_ conditional mutant locus [(i)ΔCDC48_AP_; see Fig. S1A in the supplemental material]. PCR analyses were performed to determine whether the endogenous promoter was indeed replaced with the regulatable t7s4 promoter in the (i)ΔCDC48_AP_ line; experiments with the (i)ΔCDC48_AP_ line amplified the t7s4 promoter while showing loss of the endogenous promoter (Fig. S1B). In this mutant line, CDC48_AP_ gene expression was ablated upon the addition of anhydrous tetracycline (ATc). Western blot analyses revealed that levels of the larger precursor band or CDC48_AP_ dropped below the detection limit after a single day of ATc treatment whereas the proteolytically processed mature form of CDC48_AP_ was lost after 3 days of treatment (Fig. 1A). The level of control protein remained unchanged. The loss of the unprocessed version of an apicoplast protein prior to the appearance of the mature form of the protein is typical in conditional mutants, as the mature forms of apicoplast proteins remain in a steady state in the apicoplast whereas the unprocessed forms are no longer expressed or rapidly cleaved to the mature form (14).

10.1128/mBio.00950-17.1 FIG S1  Construction of conditional mutants. (A) Graphical representation of the fosmid recombineering strategy used to construct conditional mutants. The bacterial selection marker gentamicin (Gent) is shown in orange, the *T. gondii* selection marker dihydrofolate reductase (DHFR) in red, and the conditional tetracycline promoter (t7s4) in light blue. (B) Diagnostic PCR to confirm the correct replacement of the endogenous PUBL and CDC48_AP_ promoter with the t7s4 promoter. (i)ΔPUBL and the parental ΔKu80/TATi line (P) were used in lanes 2 and 3, respectively, with a primer set that amplifies 2.6 kb of the t7s4 promoter (P1 and P2). Lanes 4 and 5 correspond to the use of primers that amplify 1 kb of the native PUBL promoter (P4 and P5). (i)ΔCDC48_AP_ and ΔKu80/TATi (P) were used in lanes 8 and 9, respectively, with a primer set that amplifies 2.6 kb of the t7s4 promoter (P1 and P3). Lanes 10 and 11 were used with primers designed to amplify 2 kb of the CDC48_AP_ promoter (P6 and P7). We observed a PCR product of the correct size for the (i)ΔPUBL and (i)ΔCDC48_AP_ lines with the t7s4 primers but witnessed no PCR product for the endogenous promoter, which suggests that the mutant promoters were correctly disrupted. All primers used are listed in Table S1. Download FIG S1, TIF file, 15.1 MB. Copyright © 2017 Fellows et al. 2017 Fellows et al. This content is distributed under the terms of the Creative Commons Attribution 4.0 International license.

The development of a conditional mutant allowed us to test whether CDC48_AP_ is necessary for parasite survival. We conducted plaque assays to examine the importance of the protein. In the absence of ATc, (i)ΔCDC48_AP_ parasites invaded host cells, replicated, and egressed, which resulted in plaque formation. However, in the presence of ATc, no plaques formed, suggesting that CDC48_AP_ is critical for parasite growth (Fig. 1B). We constructed a (i)ΔCDC48_AP_ strain expressing an ectopic copy of CDC48_AP_ tagged with an epitope tag (CDC48_AP_-myc) and repeated the plaque assay. The complemented mutant line was able to form plaques even upon the addition of ATc, demonstrating that the loss of plaque formation of the (i)ΔCDC48_AP_ line is directly linked to the loss of CDC48_AP_. CDC48 has two ATPase domains, and each contains a Walker A motif (GXXXXGKT/S, where X is any amino acid) and a Walker B motif (HHHHDE, where H is a hydrophobic amino acid) for ATP binding and hydrolysis, respectively; both are critical to the role of the protein as part of the ERAD system (26). Our annotation of the apicoplast CDC48_AP_ shows homologous ATPase domains with Walker motifs (Fig. S2A and B). We constructed point mutations in the critical residues (502 K/A and 829 E/Q). In transient transfections, mutant proteins properly localized to the apicoplast (Fig. S2C and D). However, we consistently failed to isolate stable transgenic parasites, suggesting a strong dominant-negative effect of these mutations.

10.1128/mBio.00950-17.2 FIG S2  CDC48_AP_ D1 and D2 ATPase domains and N-terminal domain. Data represent alignment of *Toxoplasma gondii* CDC48_AP_ with CDC48 of *Saccharomyces cerevisiae*. Domains were identified using the blastp algorithm from the National Center for Biotechnology Information. Domains were then aligned using Clustal Omega. Amino acids in the alignment that show identity are boxed in dark gray, while light gray indicates conservative substitutions. (A and B) Partial alignment of the D1 and D2 ATPase domains between CDC48_AP_ and *S. cerevisiae* CDC48, respectively. The Walker A motifs are highlighted in yellow, and the Walker B motifs are highlighted in red. (C and D) Immunofluorescence assay of the ΔCDC48_AP_ line expressing an ectopic myc-tagged version of CDC48_AP_ (green) with point mutations to the Walker A (ΔCDC48_AP_ K/A-myc) and Walker B (ΔCDC48_AP_ E/Q-myc) motifs. The apicoplast luminal marker chaperonin 60 (CPN60) is shown in red. Images demonstrate that ectopic CDC48_AP_ with point mutations localized to the apicoplast. (E) Alignment of the N-terminal domain of CDC48_AP_ and that of *S. cerevisiae* CDC48, which is a known ubiquitin and chaperone binding site. Download FIG S2, TIF file, 5.2 MB. Copyright © 2017 Fellows et al. 2017 Fellows et al. This content is distributed under the terms of the Creative Commons Attribution 4.0 International license.

We next tested the ability of the (i)ΔCDC48_AP_ line to import apicoplast proteins in the presence and absence of ATc. The majority of nucleus-encoded apicoplast proteins have an N-terminal bipartite leader peptide which consists of a signal peptide followed by a transit peptide (27). The transit peptide, which directs the protein to the apicoplast, is cleaved by an unknown protease in the lumen of the apicoplast; proteins that remain in the periphery show similar maturation that likely depends on the presence of a peripheral maturase. Western blot analysis of apicoplast proteins thus often results in two bands. The larger band represents the apicoplast protein en route to the organelle, and the smaller band represents the mature protein that has been processed in the lumen of the apicoplast. The loss of apicoplast import results in the loss of this processing of apicoplast protein, and we have previously exploited this to measure apicoplast import in several mutants (13, 14, 16, 28).

Acyl carrier protein (ACP), a protein that targets to the apicoplast lumen, was endogenously tagged with yellow fluorescent protein (YFP) in the (i)ΔCDC48_AP_ line (Fig. 1C). The parasite strain was grown for 0 to 3 days under ATc treatment conditions and was then harvested for Western blot analysis. The mature form of the protein was lost after 3 days of ATc treatment, while the level of the control and larger precursor band remained unchanged throughout the experiment (Fig. 1E). This suggests that the protein does not reach the lumen for processing and that parasites lacking CDC48_AP_ thus display an apicoplast import defect. To test whether CDC48_AP_ is also important for the import of the apicoplast proteins that reside in the periphery of the organelle, we followed the protein encoded by *T. gondii* ME49_201270 (TGME49_201270) (29). The transit peptide of the peripheral apicoplast protein encoded by TGME49_201270 is cleaved in the periplastid compartment (PPC) after import across the periplastid membrane (PPM) rather than in the lumen of the apicoplast (11, 29). *T. gondii* Me49_201270 (TgMe49_201270) was endogenously tagged with a hemagglutinin (HA) epitope tag in the (i)ΔCDC48_AP_ parasite line (Fig. 1D), grown under ATc treatment conditions, and analyzed by Western blotting. Treatment with ATc resulted in the loss of the mature form of the protein after 2 days (Fig. 1F). The loss of processing of peripheral and luminal apicoplast proteins suggests that (i)ΔCDC48_AP_ acts early in protein import, which is consistent with its site of residence and activity in the PPC rather than the lumen. To control for the possibility that loss of the mature form of import reporters may be due to loss of the organelle as a consequence of a broader role of CDC48_AP_ in apicoplast biology, we monitored the numbers of apicoplasts in the (i)ΔCDC48_AP_ strain under knockdown conditions. We enumerated organelles after immunofluorescence staining, and we used quantitative PCR (qPCR) to measure the relative abundance of the organellar genome (Fig. 1G; Fig. S3 and S4A). There was no significant decrease in apicoplast numbers in the (i)ΔCDC48_AP_ line until day 4 or day 5 of ATc treatment; in contrast, protein import was already affected on day 2 and day 3, arguing for a direct role of CDC48_AP_ in apicoplast protein import. No other changes in plastid morphology or marker distribution were observed in the mutant line, and the parental line showed no apicoplast biogenesis defects upon the addition of ATc (Fig. 1H).

10.1128/mBio.00950-17.3 FIG S3  Measurement of apicoplast loss through immunofluorescence. (A) Representative images of progressive apicoplast loss in mutant ΔCDC48_AP_ following ATc treatment. Quantification is shown in Fig. 1G. Apicoplasts were detected by IFA using an antibody to CPN60 (shown in red). Download FIG S3, TIF file, 10.7 MB. Copyright © 2017 Fellows et al. 2017 Fellows et al. This content is distributed under the terms of the Creative Commons Attribution 4.0 International license.

10.1128/mBio.00950-17.4 FIG S4  Apicoplast quantification through qPCR measurement of relative abundances of nuclear and plastid genomic DNA. Apicoplasts were quantified for the ΔCDC48_AP_ (A) and the ΔPUBL (B) lines, respectively, by comparison of the abundances of nuclear and apicoplast genome DNA through qPCR. Note that there was a significant loss of plastid genome only after prolonged ATc treatment. Download FIG S4, TIF file, 1.8 MB. Copyright © 2017 Fellows et al. 2017 Fellows et al. This content is distributed under the terms of the Creative Commons Attribution 4.0 International license.

### A novel ubiquitin-like protein is localized to the apicoplast.

We have previously demonstrated the importance of the ubiquitin conjugating enzyme E2_AP_ in the PPC of the apicoplast in *T. gondii* (16). A conditional mutant for this enzyme resulted in apicoplast import defects similar to those seen here for CDC48_AP_. The ubiquitination machinery and CDC48_AP_ likely work together in the PPC to import proteins. CDC48 and its many cofactors have ubiquitin binding domains which have been shown to bind and interact with polyubiquitin and monoubiquitin (19, 20, 24, 30). CDC48_AP_ retained the conserved N domain with its double-psi β-barrel motif, which is a known ubiquitin interaction site (Fig. S2E). So far, however, we have been unable to demonstrate import of cytoplasmic ubiquitin into the apicoplast or the presence of a specific apicoplast-targeted ubiquitin. Therefore, we broadened our search for an apicoplast-specific modifier to include ubiquitin-like proteins by using BLAST searches to identify proteins with a putative signal peptide and a ubiquitin-like domain; we also systematically reevaluated the gene models and transcription start sites of all ubiquitin-like genes. This effort yielded gene TgMe49_223125, which is predicted to encode a protein with a C-terminal domain with similarity to ubiquitin. For brevity, here we refer to the novel ubiquitin encoded by TgMe49_223125 as plastid ubiquitin-like protein (PUBL).

**FIG 2:**
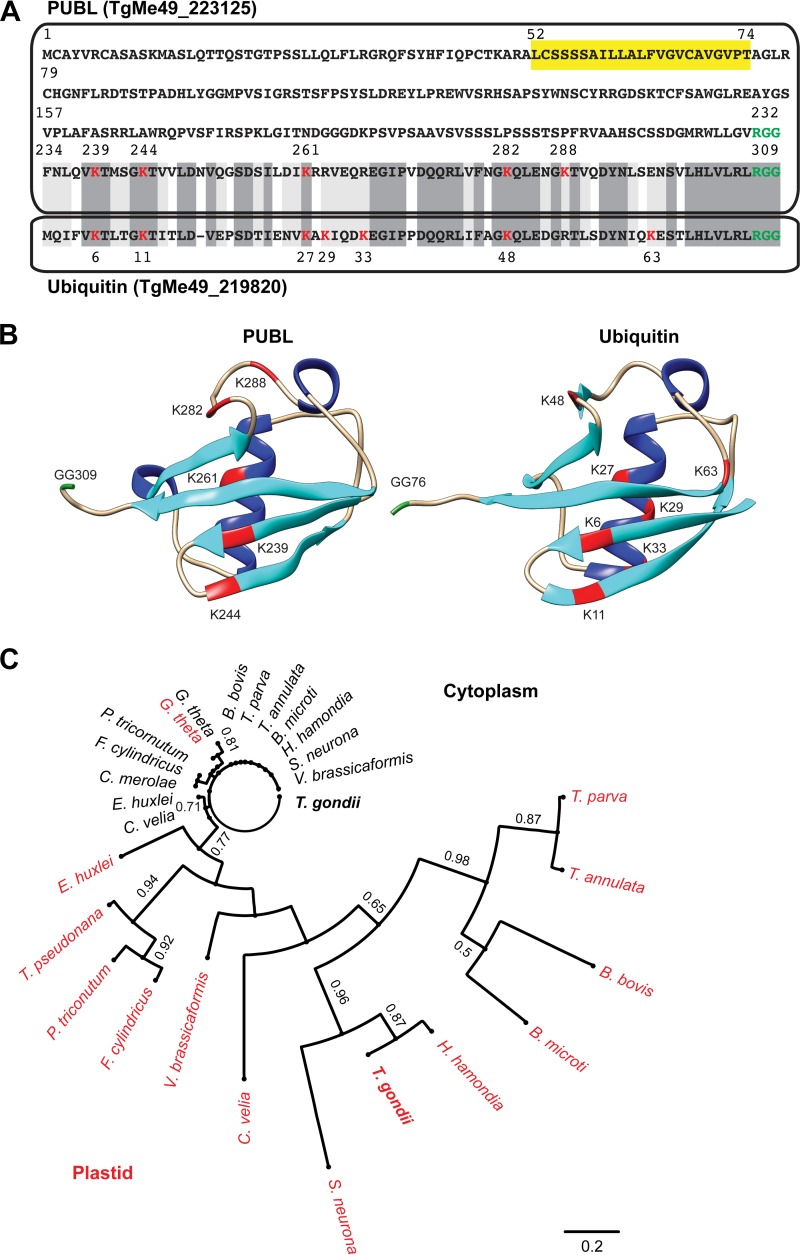
The C-terminal domain of PUBL resembles that of ubiquitin. (A) Sequence alignment of *T. gondii* PUBL and ubiquitin. A sequence that could serve as a transmembrane domain or as a recessed signal peptide is highlighted in yellow, the lysine residues of the ubiquitin domain are indicated in red, and potential deubiquitinase cleavage RGG motifs are shown in green. Lower box, *T. gondii* cytoplasmic ubiquitin. Amino acids in the alignment that show identity are boxed in dark gray, while light gray indicates conservative substitutions. (B) Ribbon diagram representation of the structure of the C-terminal domain of PUBL predicted *de novo* using Quark compared to the experimentally established structure of human ubiquitin (MMDB ID, 57540; PDB ID, 1UBQ). Beta sheets are colored cyan, alpha helices blue, lysines red, and the diglycine motif green. (C) Maximum likelihood tree depicting the phylogenetic relationship of PUBL and selected plastid (red) and cytoplasmic (black) homologs. Bootstrap values are shown for 100 replicates (for apicomplexans, *Babesia microti* [BBM_I02580 and BBM_III01010] and *B. bovis* [BBOV_III010050 and BBOV_1V010030], *Chromera velia* [CVEL_26518 and CVEL 26884], *Hammondia hammondi* [HHA_223125 and HHA_289750], *Sarcocystis neurona* [SRCN_6530 and SRCN_6527], *Theileria annulata* [TA11575 and TA16165], *Toxoplasma gondii* [TgME49_223125 and TgME49_219820], *Theileria parva* [TP02_0142 and TP01_1070], and *Vitrella brassicaformis* [Vbra_4789 and Vbra_15758]; for cryptophytes, *Guillardia theta* [155024 and 152873]; for diatoms, *Fragilariopsis cylindrus* [270635 and 2686161], *Phaeodactylum tricornutum* [54323 and 51931], and *Thalassiosira pseudonana* [1536 and 259049]; for haptophytes, *Emiliania huxleyi* [428400 and 349903]; for rhodophyta, *Cyanidioschyzon merolae* [CMK296C]).

PUBL is 311 amino acids long and is thus considerably larger than the 76-amino-acid ubiquitin found in the cytoplasm of *T. gondii*. A sequence alignment between PUBL and *T. gondii* ubiquitin revealed a C-terminal ubiquitin-like domain with 56% identity (Fig. 2A). Note for comparison that *T. gondii* ubiquitin and human ubiquitin differ by a single residue. However, using *de novo* protein structure prediction algorithms, we readily discerned the ability of PUBL to form the beta-grasp fold (Fig. 2B), which consists of two beta sheets, an alpha helix, and three additional beta sheets characteristic of ubiquitin and ubiquitin-like proteins (XP_018638338.1) (31). The structure of human ubiquitin is shown for comparison, and we note a high degree of similarity despite significant differences in the primary sequences. We identified putative homologs of PUBL in numerous apicomplexans, and, similarly to PUBL, these proteins carry N-terminal extensions (Fig. S5 and S6; also see reference 18). Surprisingly, we did not identify a close homolog in *Plasmodium*. In contrast, the lack of a PUBL homolog in the *Cryptosporidium* species was expected, as these species lack an apicoplast. Figure 2C shows a phylogenetic tree of PUBL and homologs with identifiable leader peptides from a selection of organisms with a secondary red algal plastid. The homolog of PUBL in *P. tricornutum* has been previously experimentally validated to be targeted to the plastid (32). Note that, while cytoplasmic ubiquitins are extremely conserved, plastid homologs show variations. We conclude on the basis of their similarity in sequence and structure that PUBL and homologs are likely derived from ubiquitin.

10.1128/mBio.00950-17.5 FIG S5  Alignment of ubiquitin domains of PUBL homologs identified in red alga-derived plastid-containing organisms. (A) Alignment of multiple ubiquitin domains of putative PUBL proteins in apicomplexan parasites and related organisms. Homologs were identified by a BLAST search using the amino acid sequence of *T. gondii* PUBL. Species used in the alignment included *Toxoplasma gondii* (Tg), *Hammondia hammondi* (Hh), *Sarcocystis neurona* (Sn), *Theileria annulata* (Ta), *Theileria parva* (Tpa), *Babesia microti* (Bm), *Babesia bovis* (Bb), *Phaeodactylum tricornutum* (Pt), *Thalassiosira pseudonana* (Tp), *Fragilariopsis cylindrus* (Fc), *Emiliania* *huxleyi* (Eh), *Vitrella brassicaformis* (Vb), and *Chromera velia* (Cv). Highlights are as detailed in the Fig. S2 legend. Gene IDs of putative PUBL homologs used in alignment: BBOV_III010050, SRCN_6530, HHA_223125, BBM_I02580, TA11575, TP02_0142, and 54323 for *P. tricornutum*, 270635 for *F. cylindrus*, 428400 for *E. huxleyi*, 1536 for *T. pseudonana*, and CVEL_26518 and Vbra_4789 for *V. brassicaformis*. Note that all gene products have a putative leader sequence and additional amino acids following the C-terminal “RGG” motif. Download FIG S5, TIF file, 3.8 MB. Copyright © 2017 Fellows et al. 2017 Fellows et al. This content is distributed under the terms of the Creative Commons Attribution 4.0 International license.

10.1128/mBio.00950-17.6 FIG S6  Full-sequence alignment of PUBL homologs. The species used in this alignment are the same as those used for Fig. S5 except that the N-terminal leader region is included in the alignment. No sequence conservation is seen between the homologous N-terminal regions. The “RGG” motif is colored in green. Note that most sequences have this “RGG” motif immediately before the C-terminal ubiquitin domain. Download FIG S6, TIF file, 7 MB. Copyright © 2017 Fellows et al. 2017 Fellows et al. This content is distributed under the terms of the Creative Commons Attribution 4.0 International license.

**FIG 3:**
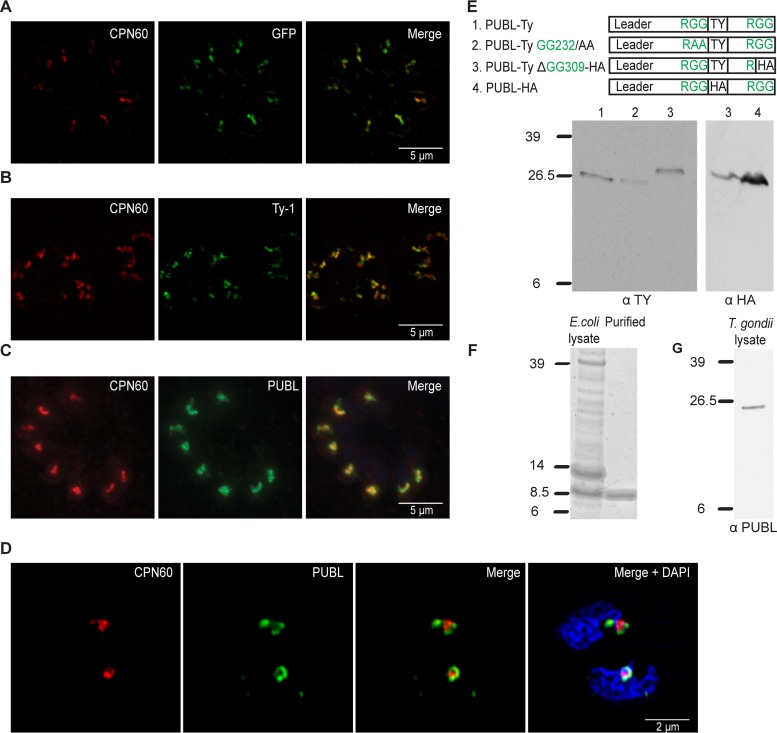
PUBL is an apicoplast-specific ubiquitin-like protein. (A) Immunofluorescence assays depicting parasites expressing the N-terminal 180 amino acids of PUBL fused to GFP (anti-GFP, green). (B) Full-length version of PUBL with an internal Ty-1 epitope tag (PUBL-Ty1, as shown in Fig. 3E-1; anti-Ty1, green). (C) ΔKu80/TATi parental parasites stained with a monoclonal antibody raised against the ubiquitin-like domain of PUBL (green). Counterstaining for CPN60 is shown in red. (D) Superresolution microscopy performed on ΔKu80/TATi parental parasites stained with PUBL antibody (green) and counterstained for CPN60 (red). (E) The first panel depicts four constructs transfected into parasite lines to express tagged and/or mutated versions of PUBL. Lanes are identified in the key at the top of the panel. Data represent the results of Western blot analysis of the protein lysates prepared from the four different lines as numerically indicated in the first panel. Note the additional HA epitope tag in construct 3, resulting in a small increase of the apparent molecular mass compared to constructs 1 and 2. (F) Coomassie-stained protein gel of protein extract and purified protein derived from *E. coli* cell expressing a recombinant version of the C-terminal ubiquitin-like domain of PUBL. An 8.5-kDa band is visible and matches the expected size of recombinant PUBL carrying a 6×His tag. (G) Western blot analysis of protein extracts of ΔKu80/TATi parasites stained with monoclonal antibody obtained through immunization with the recombinant protein as described for panel F. Note that the single bands in panels E and G are considerably larger than the 8.5-kDa band shown in panel F; slight differences in apparent molecular masses are due to use of the various epitope tags.

The reason that we overlooked this protein previously is that it is not recognized by the algorithms typically used to detect apicoplast proteins due to the lack of an N-terminal signal peptide. However, PUBL contains a predicted transmembrane domain from amino acid 52 to 74, and we considered that this portion of the protein might serve as a recessed signal peptide (Fig. 2A). We expressed tagged versions as transgenes to localize the protein. First, we tested whether the N terminus of PUBL was capable of directing apicoplast import by expressing the first 180 amino acids with a GFP tag in *T. gondii*. The immunofluorescence assay (IFA) showed that GFP colocalized with apicoplast marker CPN60 (14), demonstrating that the N terminus of PUBL acts as an apicoplast leader (Fig. 3A). Next, we wanted to tag the entire protein with an epitope. This was a complex procedure, as tagging the N terminus would likely have interfered with the N-terminal apicoplast trafficking information. Conversely, tagging ubiquitin-like proteins at the C terminus is not practical either, as this often results in the removal of the epitope tag by deubiquitinases and interferes with ubiquitination (33). We therefore amplified the coding sequence of PUBL from cDNA by PCR and ligated it into an expression plasmid in a way that introduced an internal Ty-1 epitope tag (Fig. S7A and S7B). An immunofluorescence assay was performed on a parasite line expressing the tagged protein, which showed a single punctate structure per cell. We counterstained with an antibody to CPN60, which produced labeling that overlapped PUBL, suggesting that PUBL is localized to the apicoplast (Fig. 3B). To validate this assignment independently of transgene overexpression, we expressed and purified the ubiquitin-like domain of PUBL in bacteria and raised monoclonal antibodies (Fig. 3F). Immunofluorescence assays using this new antibody again produced apicoplast labeling (Fig. 3C). While PUBL clearly is localized to the apicoplast, we observed slight differences in staining of these reagents compared to CPN60. We had previously noted comparable differences for proteins localized to the PPC (14, 16, 29). Structure illumination superresolution microscopy was performed and revealed PUBL staining surrounding the label for the innermost compartment CPN60 marker (Fig. 3D). While we cannot fully resolve the four membranes, the images are consistent with residence of PUBL in the PPC of the apicoplast alongside the previously characterized ubiquitinating machinery. Overall, we concluded that PUBL is a novel ubiquitin-like protein found in the periphery of the apicoplast and is conserved among apicomplexans.

10.1128/mBio.00950-17.7 FIG S7  Construction of internally Ty-1-tagged PUBL. (A) Diagram of the procedure used for internally tagging the PUBL coding sequence. Two initial PCRs were implemented to amplify the 5′ end (730 amino acids) and the 3′ end (273 amino acids) of PUBL. The PCR products were used as a template for a third PCR, which resulted in the final internally Ty-1-tagged PUBL gene. (B) Agarose gel of PCR products. Lane 2 represents the result of amplification of the 5′ end of PUBL. Lane 3 represents the result of amplification of the 3′ end of PUBL. Lane 6 represents the result of the final PCR amplification after the insertion into a pDC vector. Download FIG S7, TIF file, 10 MB. Copyright © 2017 Fellows et al. 2017 Fellows et al. This content is distributed under the terms of the Creative Commons Attribution 4.0 International license.

As has been pointed out, most apicoplast proteins are proteolytically processed to remove the transit peptide. In addition, PUBL has an RGG motif that immediately precedes its C-terminal ubiquitin-like domain. Deubiquitinases typically cleave polyubiquitin and other precursors at this position, releasing the 76-amino-acid ubiquitin domain (34). On the basis of this precedent, we expected the size of mature PUBL to be 8.5 kDa, similar to the size of ubiquitin. Multiple tagged versions of PUBL were generated to test this hypothesis, including a form that mutated the two glycine residues at amino acid position 232 to alanine, which should prevent cleavage (Fig. S8E). However, Western blot analysis revealed a single 26.5-kDa or 29.5-kDa band corresponding to the full-length PUBL protein for every version of the epitope-tagged PUBL (Fig. 3E). This suggests a lack of processing in PUBL. It is conceivable that we might fail to detect mature PUBL due to folding or steric hindrance or that epitope tagging blocks processing. However, we note that this was an observation that was highly reproducible not only using a variety of epitope tags and tagging positions within the protein but also using native protein detected by our new antibody (Fig. 3G).

10.1128/mBio.00950-17.8 FIG S8  Immunofluorescence assay of (i)ΔPUBL expressing ectopic mutated versions of epitope-tagged PUBL. (A to E) Immunofluorescence assays were performed on the stable complemented (i)ΔPUBL lines PUBL-Ty K239/R, PUBL-Ty K244/R, PUBL-Ty K282/R, PUBL-Ty K288/R, and PUBL-Ty GG232/AA, respectively. All ectopic versions of PUBL were tagged internally with the Ty-1 epitope (green), while CPN60 (red) was used as an apicoplast marker. Correct localization of the ectopic PUBL in the apicoplast was observed for all mutants. Download FIG S8, TIF file, 3.8 MB. Copyright © 2017 Fellows et al. 2017 Fellows et al. This content is distributed under the terms of the Creative Commons Attribution 4.0 International license.

### PUBL is essential for parasite survival and import across the PPM of the apicoplast.

**FIG 4:**
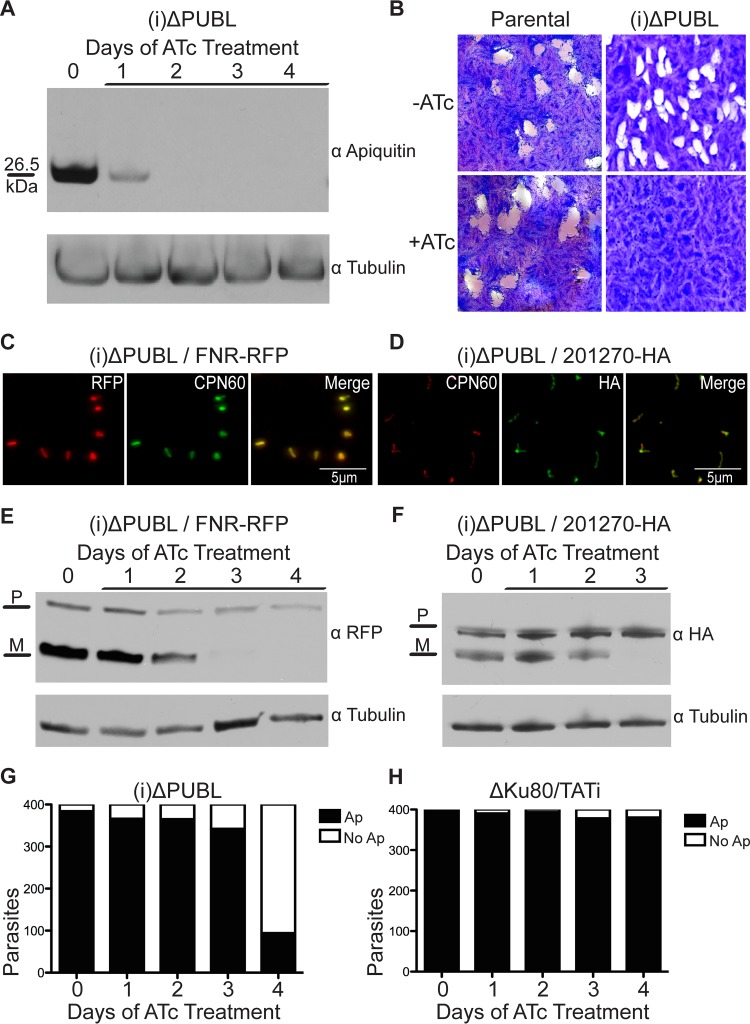
Loss of PUBL leads to loss of protein import across the PPM and a block of parasite growth. (A) Western blot analysis of (i)ΔPUBL parasites grown under ATc treatment conditions for the indicated times. (B) Plaque assay measuring growth of the (i)ΔPUBL line in the presence and absence of ATc. (C and D) Immunofluorescence assay performed on stable (i)ΔPUBL lines expressing FNR-RFP (red) (C) and 201270-HA (green) (D). CPN60 is shown as an apicoplast marker. No change in the localization of these two proteins was visible after the first 4 days of ATc treatment. (E and F) Apicoplast import assays were performed on the (i)ΔPUBL/FNR-RFP (E) and (i)ΔPUBL/201270-HA (F) lines. Note the loss of the mature band (M) under ATc treatment conditions, while the precursor band (P) remained unchanged. (G) The presence of apicoplast was scored by IFA for the indicated times of ATc treatment as detailed for Fig. 1 for the CDC48_AP_ mutant. Note that representative micrographs are shown in Fig. S3 and independent measurements of plastid loss following the organellar genome in Fig. S4B. (H) The same assay was performed on the parental ΔKu80/TATi lines. Note that there was no significant difference in apicoplast numbers after ATc treatment. Anti-tubulin served as the loading control in the experiments whose results are presented in panels A, E, and F.

In order to test PUBL’s importance in the apicoplast, we constructed a conditional mutant [(i)ΔPUBL] following the strategy described for the CDC48_AP_ mutant. PCR analysis indicated that the tetracycline-regulated promoter replaced the endogenous promoter in this PUBL mutant (Fig. S1B), and Western blot experiments showed that PUBL was no longer detectable after the second day of ATc treatment (Fig. 4A). We performed plaque assays with the (i)ΔPUBL line. Addition of ATc to the mutant strain resulted in the loss of plaque formation (Fig. 4B), demonstrating that PUBL is required for parasite growth.

Previous work showed that the PPC ubiquitinating machinery is crucial for import of proteins into the apicoplast (16). We thus asked whether PUBL would also be essential for import. Ferredoxin NADPH reductase (FNR) is a luminal apicoplast protein which we are able to tag with red fluorescent protein (35). A stable line was isolated that ectopically expressed FNR-red fluorescent protein (FNR-RFP) in the PUBL mutant (Fig. 4C) (for technical reasons, we were unable to engineer an ACP-YFP line in this mutant, but we note that the two markers show indistinguishable localization and targeting results [35]). This parasite line was treated with ATc for 0 to 4 days and was prepared for Western blot analysis. The smaller mature band was diminished after 2 days of ATc treatment and was lost after 3 days, while the levels of control and precursor protein remained constant (Fig. 4E). We also endogenously tagged the TgMe49_201270 peripheral apicoplast gene with an HA epitope tag in the (i)ΔPUBL line (Fig. 4D). Again, Western blot analysis showed reduced maturation after 2 days of ATc treatment and loss of the mature band after 3 days. We thus found loss of processing of luminal and peripheral proteins, which suggests that PUBL is critical for an early step of protein import into the apicoplast, likely that of translocation of the membrane that bounds the PPC. Apicoplast numbers were measured in the mutant strain via immunofluorescence loss analysis and quantitative PCR, and the results showed no significant loss of apicoplast numbers prior to loss of apicoplast protein import (Fig. 4G and H; Fig. S4B). No other morphological changes were observed in the mutant line.

### Genetic complementation of mutant reveals terminal glycines to be critical for PUBL function.

**FIG 5:**
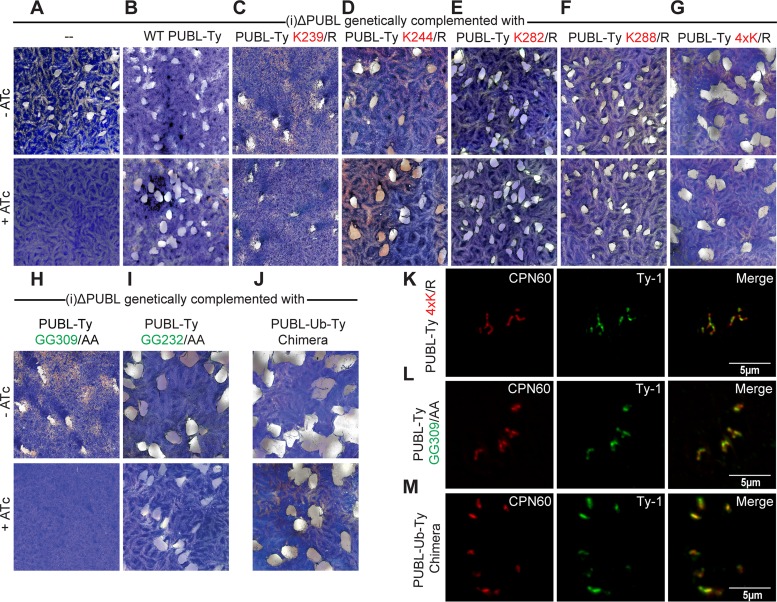
Genetic complementation analysis reveals essential PUBL residues. Wild-type sequences as well as a range of point mutations of the PUBL coding sequence (all marked with an internal Ty tag) were introduced into the UPRT locus of the (i)ΔPUBL line. (A to I) Plaque assays were performed in the absence or presence of ATc to test the ability of each mutant to complement the loss of PUBL expression from the native locus. Lysine was replaced with asparagine and glycine with alanine; mutant 4xK was mutated at all four previously indicated positions. (J) Complementation experiment was performed as described above using a parasite line that encodes a chimera in which the C-terminal domain of PUBL is replaced with the *T. gondii* ubiquitin sequence. (K to M) Immunofluorescence assays of complemented mutant (additional data shown in Fig. S5). Note that all transgenes encode proteins that show localization indistinguishable from that of the wild-type protein. CPN60 was used as an apicoplast marker for counterstaining.

We tested whether the (i)ΔPUBL line could be complemented with an ectopic copy of PUBL. We introduced an extra copy of Ty-1 epitope-tagged PUBL into the uracil-phosporibosyltransferase (UPRT) locus, disrupting UPRT function. Loss of UPRT confers resistance to 5-fluorodeoxyuridine (FUDR), which we exploited for selection. We confirmed expression and correct localization of the ectopic Ty-1 epitope-tagged PUBL through immunofluorescence assay (Fig. 5K to M; Fig. S8). This line is able to form plaques when cultured in the presence of ATc, demonstrating that the extra copy was able to rescue the growth defect of the mutant line (Fig. 5A and B). Lysine residues are of particular importance in the biology of ubiquitin as they serve as sites for linkages to form polyubiquitin chains. Different lysine chain linkages are recognized as signals for distinct biological processes (36). PUBL has only five lysine residues in the ubiquitin-like domain of the protein, with four of the lysines being shared with ubiquitin and one nonconserved lysine at position 288. We systematically mutated the five lysine residues in the ubiquitin domain to the similarly charged arginine residue. Previous research showed that mutating lysine to arginine prevents the formation of polyubiquitin lysine-linked chains (37). Mutations to the lysines at positions 239, 244, 282, and 288 of the ubiquitin domain of Ty-1 epitope-tagged PUBL were engineered. These extra copies of PUBL were introduced into the UPRT locus of the (i)ΔPUBL line. Plaque assays were performed with these lines, and expression of the mutated PUBL was able to complement the (i)ΔPUBL line for all lysine mutations (Fig. 5C to F), suggesting that poly-PUBL does not form or is not important. We considered that PUBL might utilize lysine residues in a redundant fashion and that point mutations to single lysine residue thus would not affect poly-PUBL chain formation. Therefore, we introduced into the (i)ΔPUBL line a tagged PUBL which contained all four lysine point mutations. This line was able to fully complement the loss of endogenous PUBL (Fig. 5G). The experiment suggests that these four lysine residues are not critical for PUBL’s function in the apicoplast. We note that we were unable to establish a stable line expressing a point mutation at the lysine at position 261 of the ubiquitin domain, suggesting a dominant-negative effect of this mutation. We were thus unable to test the importance of this residue.

One of the key features of ubiquitin and ubiquitin-like proteins is the ability to bind to other proteins via a C-terminal glycine, and PUBL shares the requisite diglycine motif (38) (see Fig. 2A). It is well documented that mutating these glycines to alanine residues results in conjugation-deficient ubiquitin and ubiquitin-like protein (39, 40). We examined the importance of such conjugation by introducing a PUBL with mutated C-terminal glycines into the (i)ΔPUBL line. This mutant was unable to complement the (i)ΔPUBL line (Fig. 5H). We thus conclude that the PUBL C-terminal diglycine motif is indispensable for its function, indicating that PUBL transferred to substrate proteins in manner similar to that seen with ubiquitination. As discussed previously, PUBL harbors a second diglycine motif immediately preceding the ubiquitin-like domain. Mutation of these residues did not affect complementation (Fig. 5I). This is consistent with our observation of a lack of processing at this site. We hypothesize that PUBL acts like ubiquitin and is transferred onto proteins. PUBL diverged from ubiquitin considerably but retained the stereotypical ubiquitin fold. We therefore tested whether ubiquitin, when appropriately localized, could rescue the loss of PUBL. An expression vector was engineered which replaced the ubiquitin domain of PUBL with *T. gondii* ubiquitin. As shown in Fig. 5J, expression of the PUBL/ubiquitin chimera in the (i)ΔPUBL line (PUBL-UB-Ty Chimera) resulted in the ability of the line to form plaques under ATc treatment conditions. We noted a slight but consistent reduction of plaque size for this strain upon addition of ATc (Fig. 5J). We interpret this result to indicate that PUBL acts in a fashion highly similar to the activity of ubiquitin and that replacement of PUBL with ubiquitin thus produces only minor attenuation of that function.

## DISCUSSION

It is difficult to overrate the importance of endosymbiosis in the early evolution of eukaryotes. Many (but certainly not all) evolutionary biologists have come to view the initial endosymbiotic acquisition of an alpha-proteobacterium not only as the point of origin of the mitochondria but also as the very birth of the eukaryotic cell. Chloroplasts had a similar endosymbiotic genesis; this event harnessed the ability to photosynthesize and allowed eukaryotes to conquer the problem of primary production. Since then, a series of secondary and tertiary events of uptake and loss has given rise to tremendous organismic diversity. Horizontal gene transfer from the newly acquired organelle to the host nucleus is a hallmark of all endosymbiosis events (41). This process endows the host with control over its symbiont but also requires the concurrent evolution of posttranslational mechanisms to route symbiont proteins now encoded and produced by the host into the organelle. For the mitochondrion and primary chloroplasts, specific cargo recognition and translocation complexes facilitate protein translocation across each of the membranes of the respective organelle and this process is understood in considerable mechanistic detail (42, 43). *Apicomplexa* and other phyla, including those encompassing chromera, dinoflagellates, haptophytes, cryptophytes, and diatoms, possess a secondary plastid of red algal origin (44, 45). Consistent with its evolutionary origin, the apicoplast shares import machinery with primary plastids, specifically, the TOC complex and the TIC complex, which facilitate protein import across the outer and inner membranes of the chloroplast. In previous studies, we demonstrated that the *T. gondii* apicoplast relies on a homolog of Toc75 for protein import across the second innermost membrane whereas Tic20 and Tic22 homologs are required for import across the innermost membrane (11–13). The apicoplast is surrounded by four membranes, and nucleus-encoded proteins thus have to cross two membranes before they encounter the TOC complex.

Endosomal and autophagic pathways are candidate mechanisms that may provide guidance to and fuse vesicles with the outermost apicoplast membrane. Intriguingly, autophagy-related protein 8 and phosphatidylinositol 3-phosphate heavily accumulate on the surface of the apicoplast. However, genetic interference with these mechanisms in *T. gondii* and *Plasmodium falciparum* did not produce an unequivocal link to protein import but rather pointed to a broader role of autophagy in apicoplast morphogenesis and inheritance (46–48). While the nature of transport across the outermost membrane remains to be revealed, we know more about the PPM that imported proteins encounter next. Pioneering observations by Maier and colleagues in cryptomonads led to a model in which the symbiont’s ERAD pathway was retooled to serve as a mechanism of import into the periplastid space—the former cytoplasm of the red alga (18, 32, 49–52). This model received robust experimental support from genetic studies in *T. gondii* that demonstrated the requirement of the ERAD components Der1 and ubiquitin conjugating enzyme for apicoplast protein import (14, 16). However, not all secondary plastids utilize such ERAD-derived proteins. Secondary plastids derived from endosymbiosis of green algae appear to rely for import across the PPM on a mechanism that is independent of ERAD and that has yet to be fully characterized (53).

In the current study, we demonstrated that CDC48_AP_ is a critical component of the ERAD-derived apicoplast import machinery. Loss of the protein results in loss of import. We probed the mutant with different cargo proteins and found import of luminal proteins and import of periplastid proteins to be equally blocked. This is in contrast to observations that we recently reported for a *T. gondii* Toc75 mutant (11); in that mutant, only the import of luminal proteins was ablated. This difference genetically establishes the order of the translocons in the apicoplast. CDC48_AP_ likely acts as the motor of the translocon; point mutations in the Walker A and B motifs ablating CDC48_AP_’s ATPase function proved to be highly deleterious and were not tolerated by the parasite. The translocation activity of cytoplasmic CDC48 in the context of ERAD at the ER membrane requires ubiquitination of cargo (15, 19, 30). CDC48_AP_, like its cytoplasmic homolog, features ubiquitin binding domains. We note, however, that we were unable to document a biochemical interaction between CDC48_AP_ and PUBL by coprecipitation and did not observe changes in PUBL banding patterns under CDC48_AP_ knockdown conditions in Western blot analyses (data not shown).

There are numerous ubiquitin-like proteins recognized in eukaryotes that act in an array of diverse cellular processes, and we propose PUBL as a new member of this family (54). Ubiquitin is famous for being one of the most conserved proteins among eukaryotes; there is only a single amino acid difference between the sequences of the cytoplasmic ubiquitins of *T. gondii* and *Homo sapiens*. However, there is considerable sequence difference between *T. gondii* PUBL and ubiquitin. Similarly, the homologs of PUBL and ubiquitin that carry identifiable plastid leaders are quite diverse (Fig. 2C). The absence of a *Plasmodium* PUBL homolog is surprising. A putative ubiquitin-like protein, *P. falciparum* 3D7_081570 (PF3D7_081570), has been described as being localized to the apicoplast in *P. falciparum* (51). However, this protein lacks key features shared by PUBL homologs, including the diglycine motif typical of ubiquitin-like proteins. We believe that the divergence and diversity of PUBL reflect a relaxation of functional constraint. Ubiquitin has to interact with literally hundreds of cellular proteins to fulfill its multitude of functions; these multifaceted interactions enforce conservation. Evolution streamlined and simplified the red alga into the apicoplast, gradually reducing ubiquitin interactions, and thus released the leash on sequence conservation.

Alternatively, changes in PUBL sequence could be consequences of changes in function. We conducted genetic experiments to address this issue. Loss of PUBL was not tolerated by the parasite, as it blocks apicoplast protein import. We note that, as for CDC48_AP_, this block applied to luminal proteins and periplastid proteins, associating both with the peripheral ERAD-derived translocon. We used complementation analysis to further explore the functional relevance of specific residues. PUBL has five lysine residues that potentially could enable poly-PUBL chain formation. We replaced four of these individually and collectively with arginine, and those mutants fully complemented ATc-induced gene ablation in *trans*. The C-terminal RGG motif typically required for conjugation onto substrate protein is required for complementation, whereas the RGG preceding the ubiquitin-like domain is not. Finally, a chimera in which the C terminus of PUBL was replaced with cytoplasmic ubiquitin showed complementation. Overall, these data strongly support a model in which PUBL is conjugated via a C-terminal glycine onto other proteins in a fashion analogous to that seen with ubiquitin. This is in agreement with our previous analysis of the apicoplast ubiquitinating enzymes in *T. gondii* and *P. falciparum* (16) and with studies on the diatom *Phaeodactylum tricornutum*, which shares ancestry and a secondary plastid with apicomplexans (55). Polyubiquitin chains with specific lysine linkages have recently been identified in *T. gondii* and were shown to be recognized by specific deubiquitinases and to accumulate at different points of the cell cycle, emphasizing the complexity of polyubiquitin chains (56). Polychain formation is apparently not critical for PUBL’s function; this is fitting, as it is typically associated with protein degradation. However, we caution that a K261R mutation showed a dominant-negative effect, which limited our ability to test all residues. K261 is highly conserved in PUBL and its homologs (18) (see Fig. S5 in the supplemental material). Interestingly, proteolytic processing at the N-terminal RGG to release a “mature” ubiquitin maturation from larger precursors (38) was not required for PUBL function, which is consistent with our Western blot measurements of the molecular mass of PUBL which suggest the absence of processing (Fig. 3E and G).

What is the target of PUBL? Recent studies by Lau and colleagues highlighted the transit peptide as a likely site of cargo ubiquitination during plastid import in *P. tricornutum* (57, 58). Mutation of all lysines in this region abolished import; reintroduction of lysine in a different position of the transit peptide restored import. Revealingly, imported proteins lacking lysine residues in the leader peptide appeared “frozen” in the PPM, where they associate with a 540-kDa protein complex. This complex appears to contain Der1 homologs and may further interact with homologs of UBX, a protein that recognizes ubiquitin in the context of CDC48. Pulldown experiments with PUBL, RFP, and HA antibodies were performed using (i)ΔPUBL, (i)ΔPUBL/FNR-RFP, and (i)ΔPUBL/TgMe49_201270-HA lines, respectively, to determine whether PUBL is bound to cargo proteins. We did not observe bands in addition to those corresponding to PUBL itself in Western blot analysis and thus lack a biochemical demonstration of PUBL involvement in peptide linkage to cargo proteins in *T. gondii* (data not shown). A comprehensive study on ubiquitination in *T. gondii* highlighted the difficulty of identifying a substrate by pulldown experiments and did not generate evidence for ubiquitination or PUBL modifications of apicoplast proteins (59). To our knowledge, PUBL modifications have also not been formally demonstrated in diatoms (55). This could merely reflect the very transient nature of these modifications, which may be restricted to the translocation event itself, thus severely limiting the conjugated pool available for detection. Apicoplast-specific deubiquitinases may act swiftly to remove PUBL upon translocation; PUBL transfer and removal could even be physically linked as part of a multiprotein translocation complex. Alternatively, PUBL may have a regulatory function in import and modification of the E3 ligase could be required for translocation. Recently, it was illustrated through *in vivo* and *in vitro* experiments that autoubiquitination of lysine residues of ERAD ubiquitin ligase Hrd1p is essential for translocation of misfolded proteins across the ER membrane. This suggests that ubiquitination of the ubiquitin ligase controls when proteins are exported out of the ER lumen (23). At any rate, the adaptation of ERAD to protein import required its dissociation from protein degradation. One satisfying hypothesis to potentially explain this is that of loss of the K48 polyubiquitination site on ubiquitin that typically drives proteasome interaction, which was observed in diatoms (18, 55). However, this residue is still present in PUBL (and its apicomplexan homologs) but is dispensable for its function. A translocation complex in which ubiquitination is transient and deubiquitination is a requirement for cargo release may also protect cargo. These hypotheses could be tested by identifying and genetically ablating apicoplast deubiquitinases. Loss of the activity could result in the accumulation of PUBL-modified proteins. Such accumulation of ubiquitin modification has been observed for deubiquitinase mutants in other cellular contexts (60, 61).

We now understand PUBL to be an essential part of the apicoplast protein import machinery, but important mechanistic aspects of its addition and, particularly, removal remain to be worked out. Interference with ubiquitination and deubiquitination has emerged as a rich ground for the development of drugs targeting cancer and infection (62). Pursuing these mechanisms may serve purposes beyond those that are of obvious interest to evolutionary cell biology.

## MATERIALS AND METHODS

### Cell culture and transfection.

*T. gondii* RH and ΔKu80/TATi strains were cultivated in human foreskin fibroblasts (HFFs) in Dulbecco’s modified Eagle’s medium supplemented with fetal bovine serum, penicillin-streptomycin, and l-glutamine. Transfections were carried out by resuspending parasites in cytomix supplemented with 2 mM ATP and 5 mM glutathione to 3.3 × 10^7^ parasites per ml. A 300-µl volume of the parasite suspension and 30 µg of plasmid were mixed and transferred to a 2-mm-gap-length cuvette and electroporated using a single 1.5-kV pulse, a resistance level of 25 Ω, and a capacitor setting of 25 µF. Parasites were selected in the presence of 1 µM pyrimethamine, 20 µM chloramphenicol, or 5 µM FUDR.

### Tagging of genes and genetic complementation.

Vector pDC was constructed by replacing the tubulin promoter with *T. gondii* DHFR promoter in expression plasmid pTC (63). The coding region of PUBL (TgME49_223125) was amplified from *T. gondii* cDNA using primers that introduced a Ty-1 epitope tag immediately before the C-terminal ubiquitin domain (see Fig. S7 in the supplemental material for details). This internally tagged PUBL was inserted into vector pDC using BglII and EcoRV restriction cut sites. The vector expressing *T. gondii* cytoplasmic ubiquitin (TGME49_219820) fused to the N terminus of PUBL (PUBL/ubiquitin vector) was constructed using a similar approach (see Table S1 in the supplemental material). The CDC48_AP_ complementation vector was constructed by amplifying the coding sequence of CDC48_AP_ from cDNA and inserting the amplicon into vector pTCM_3_, which introduces a 3× myc epitope tag at the C terminus of the CDC48_AP_ protein. The peripheral apicoplast protein encoded by gene TGME49_201270 was endogenously tagged with a HAx3 tag by introducing linearized vector p3HA.LIC.CATΔpacI-201270 into parasites (29). Acyl carrier protein was endogenously tagged with a YFP tag by transfecting parasites with linearized vector pLicCATYFPΔpacI-ACP. Ferredoxin NADPH reductase tagged with RFP was introduced into the (i)ΔPUBL line and was subjected to flow cytometry with transgenics. If not stated otherwise, all transgenic parasites used here were clonal lines established by limited dilution. Complementation assays were performed by transfecting 30 µg of a Cas9 plasmid that introduces a cut in the UPRT gene along with 30 µg of a PCR product that included the gene of interest marked with an epitope tag and suitable flanks to guide insertion into the UPRT locus by homologous recombination (64). Transfectants were cultured in 5 µM FUDR to select for disruption of the UPRT gene due to insertion of the ectopic complementation cassette, and expression and proper localization of the complementing transgene following the epitope tag were tested by immunofluorescence assay (IFA; see Fig. 4K to M; also see Fig. S8). A QuikChange II site-directed mutagenesis kit (Stratagene) was used to generate point mutations using the manufacturer’s protocols and primers listed in Table S1.

10.1128/mBio.00950-17.9 TABLE S1  List of the primers used in the study and their purposes. Download TABLE S1, DOCX file, 0.02 MB. Copyright © 2017 Fellows et al. 2017 Fellows et al. This content is distributed under the terms of the Creative Commons Attribution 4.0 International license.

### Construction of conditional mutant.

Mutants were constructed by replacing the endogenous promoter with the conditional promoter in a ΔKu80/TATi parasite line. A fosmid construct was engineered as previously described to replace the promoter of the gene of interest with the tetracycline conditional t7s4 promoter (25) (see Fig. S1). The primers used are listed in Table S1. The fosmids used here were RHfos08E17 and RHfos22J15 for the development of the CDC48_AP_ and PUBL mutants, respectively. The modified fosmids were transfected into the ΔKu80/TATi strain, and pyrimethamine drug selection was used to isolate stable parasite lines. Parasite lines which successfully replaced the endogenous promoter with the conditional t7s4 promoter were identified by PCR mapping of the genomic locus of the targeted gene (see Fig. S1). Mutants were tested by plaque assay as previously described (13).

### Microscopy.

HFF coverslip cultures were infected with parasites in the absence of ATc (unless otherwise stated) and, 24 h after infection, were fixed with 4% paraformaldehyde for 20 min, blocked with 3% bovine serum albumin (BSA) for 10 min, and permeabilized with 0.2% Triton X-100–3% BSA–phosphate-buffered saline (PBS) for 20 min. The primary antibodies used were mouse anti-PUBL (generated in this study) at 1:200, rabbit anti-CPN60 (14) at 1:2,000, rat anti-HA (clone 3F10; Roche Applied Science) at 1:400, mouse anti-GFP (Torry Pines Biolab) at 1:500, and mouse anti-Ty-1 (a gift from Keith Gull, Oxford University) at 1:20. The secondary antibodies used were goat anti-mouse Alexa Fluor 488, goat anti-rat Alexa Fluor 488, goat anti-rabbit Alexa Fluor 488, and goat anti-rabbit Alexa Fluor 546 (Invitrogen) at 1:2,000. Images were collected using an Applied Precision Delta Vision microscope. Images were deconvolved and adjusted for contrast using Softworx software. Superresolution structure illumination images were acquired with a Zeiss ELYRA S1 microscope, and images were processed using Zeiss Zen software.

### Western blotting.

*T. gondii* parasites were harvested (1 × 10^6^), lysed in radioimmunoprecipitation assay (RIPA) lysis buffer, boiled in 1× NuPAGE LDS sample buffer, loaded onto precast 10% Any-KD Mini-Protean TGX gels (Bio-Rad), and run at 150 V (13). Proteins were transferred to nitrocellulose membranes and probed with antibodies mouse anti-PUBL (generated in this study) at 1:200, rabbit anti-CDC48_AP_ (14) at 1:500, mouse anti-tubulin (12G10; a gift from Jacek Gaertig, University of Georgia) at 1:2,000, rat anti-HA (clone 3F10; Roche Applied Science) at 1:400, mouse anti-GFP (Torry Pines Biolab) at 1:500, and rabbit anti-RFP (Rockland Immunochemicals) at 1:1,000 followed by incubation of the membranes with horseradish peroxidase-conjugated anti-mouse, anti-rat, or anti-rabbit antibodies (Bio-Rad) (1:10,000 dilution). Bands were detected by incubation of the membrane with Pierce ECL Western blotting substrate and exposure of the membrane to film.

### qPCR.

*T. gondii* genomic DNA was extracted and prepared from parasites in the presence or absence of ATc treatment by the use of Qiagen’s DNeasy Blood and Tissue kit. A 150-ng volume of the genomic DNA was used for the qPCRs. The qPCR was performed using SYBR green mix (Bio-Rad) and primers UPRT-qPCR-F/UPRT-qPCR-R to amplify the nuclear genome and primers Apg-qPCR-F/Apg-qPCR-R to amplify the apicoplast genome as previously described (11). Copy number control was performed by making a standard curve for each qPCR based on the serial dilution of plasmids (10^8^ copies to 10^3^ copies) containing the UPRT locus or the apicoplast genome as previously described (11). All reactions were performed in triplicate in a Bio-Rad iQ5 real-time PCR detection system. The copy numbers of the apicoplast DNA and the copy numbers of the nuclear DNA were separately normalized such that parasites grown in the absence of ATc were assigned a value of 1.

### Phylogenetic analysis and 3D protein modeling.

A maximum likelihood phylogenetic tree was constructed using the software tools offered through Phylogeny.fr (65) and was visualized using figtree. *T. gondii* ubiquitin was arbitrarily chosen to root the tree. *Ab initio* protein folding and structure predictions were generated using the Quark algorithm (66). The resulting three-dimensional (3D) protein model and the established human ubiquitin structure were visualized using UCSF Chimera (67). Sequences were aligned using default T-Coffee settings and were viewed through Jalview (68). Analysis of the conservation between sequences was performed using JABAWS (69).

### Antibody development.

The C-terminal ubiquitin-like domain was inserted into vector pAVA421 to encode a fusion protein in which the last 77 amino acids of PUBL are expressed with a 6× His tag at the N terminus. The resulting PUBL expression plasmid was transformed into BL21 *Escherichia coli* cells. Protein expression was induced with 1 mM IPTG (isopropyl-β-d-thiogalactopyranoside) at 37 C for 4 h, cells were lysed, and proteins were purified by affinity chromatography using nickel-nitrilotriacetic acid (NI-NTA) resin as previously described (70). Mice were injected with 100 µg of recombinant protein with incomplete Freund’s adjuvant. Additional booster injections of 50 µg of recombinant protein with incomplete Freund’s adjuvant were given every 2 weeks. After 8 weeks, mice were sacrificed and B cells were fused with myeloma cells. Hybridomas were tested to identify clones that expressed antibody to PUBL.

### Immunoprecipitation.

(i)ΔPUBL, (i)ΔPUBL/FNR-RFP, and (i)ΔPUBL/TgMe49_201270-HA parasite lines were used for immunoprecipitation experiments. Parasites (1 × 10^9^) were collected and washed once with 1× PBS. Parasites were lysed under various conditions (using RIPA buffer and sonication in hypotonic buffer [20 mM HEPES, 10 mM KCl, 400 mM mannitol, 2 nM EDTA]) and supplemented with Roche protease inhibitor and 10 mM N-ethylmaleimide to reach a concentration of approximately 5 × 10^8^ parasites/ml. Lysed parasites were incubated overnight at 4°C with 20 µl of PUBL, RFP, or HA antibody. A 100-µl volume of Sepharose-bound protein A or G (Santa Cruz) was added, and the reaction mixture was incubated at room temperature for an hour for rabbit or mouse antibody, respectively. Samples were washed five times in wash buffer (50 mM Tris [pH 8], 200 mM NaCl, 2 mM EDTA, 1% NP, supplemented with Roche protease inhibitor), resuspended in 100 µl of 1× NuPAGE LDS sample buffer, and boiled for 5 min. Elution fractions were analyzed through Western blotting.
