# Supplementary figures and images for: A Plastid Protein That Evolved from Ubiquitin and Is Required for Apicoplast Protein Import in Toxoplasma gondii
Source: mBio. 2017 Jun 27;8(3):e00950-17. doi: 10.1128/mBio.00950-17 (PMC5487736; doi:10.1128/mBio.00950-17)

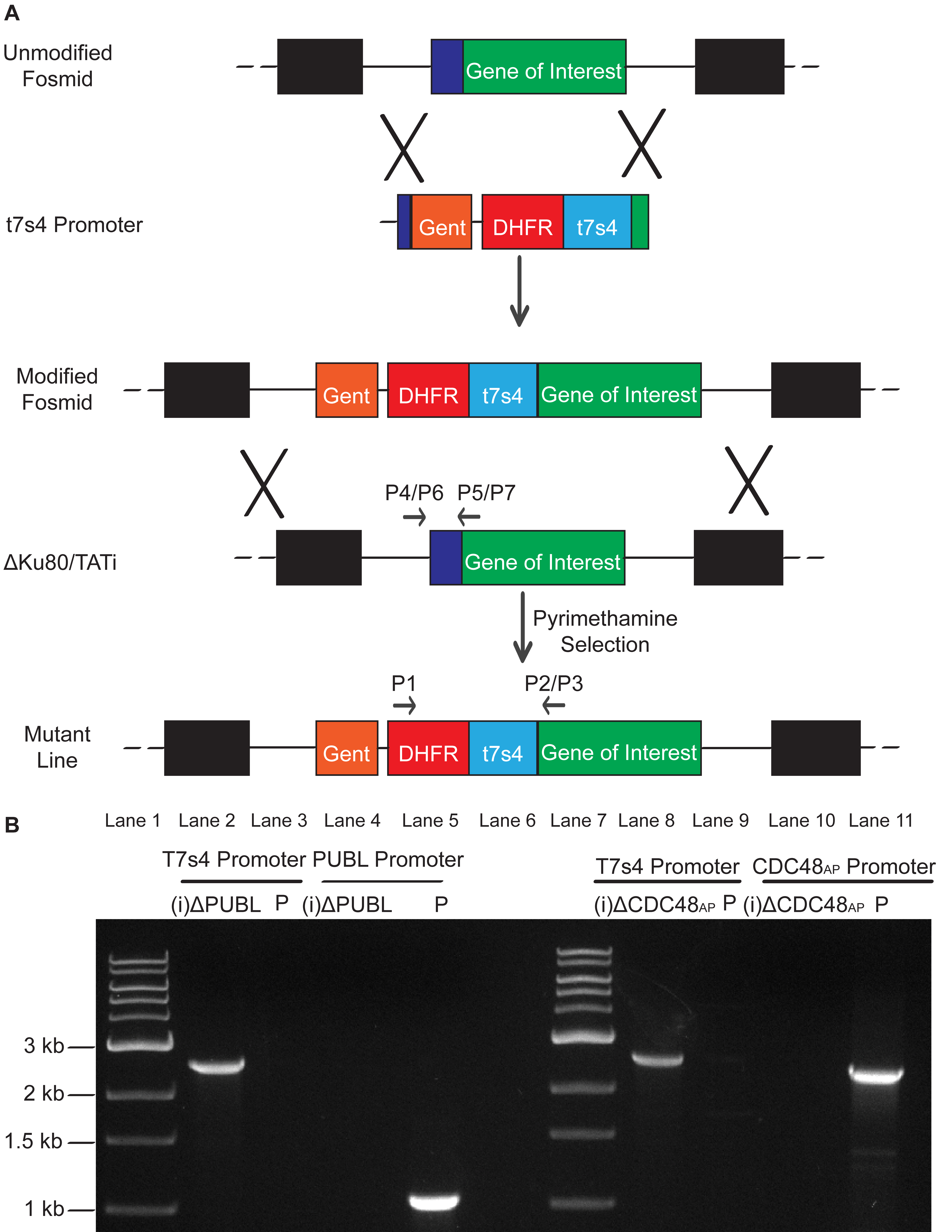

Supplement: FIG S1 [file mbo003173369sf1.tif]

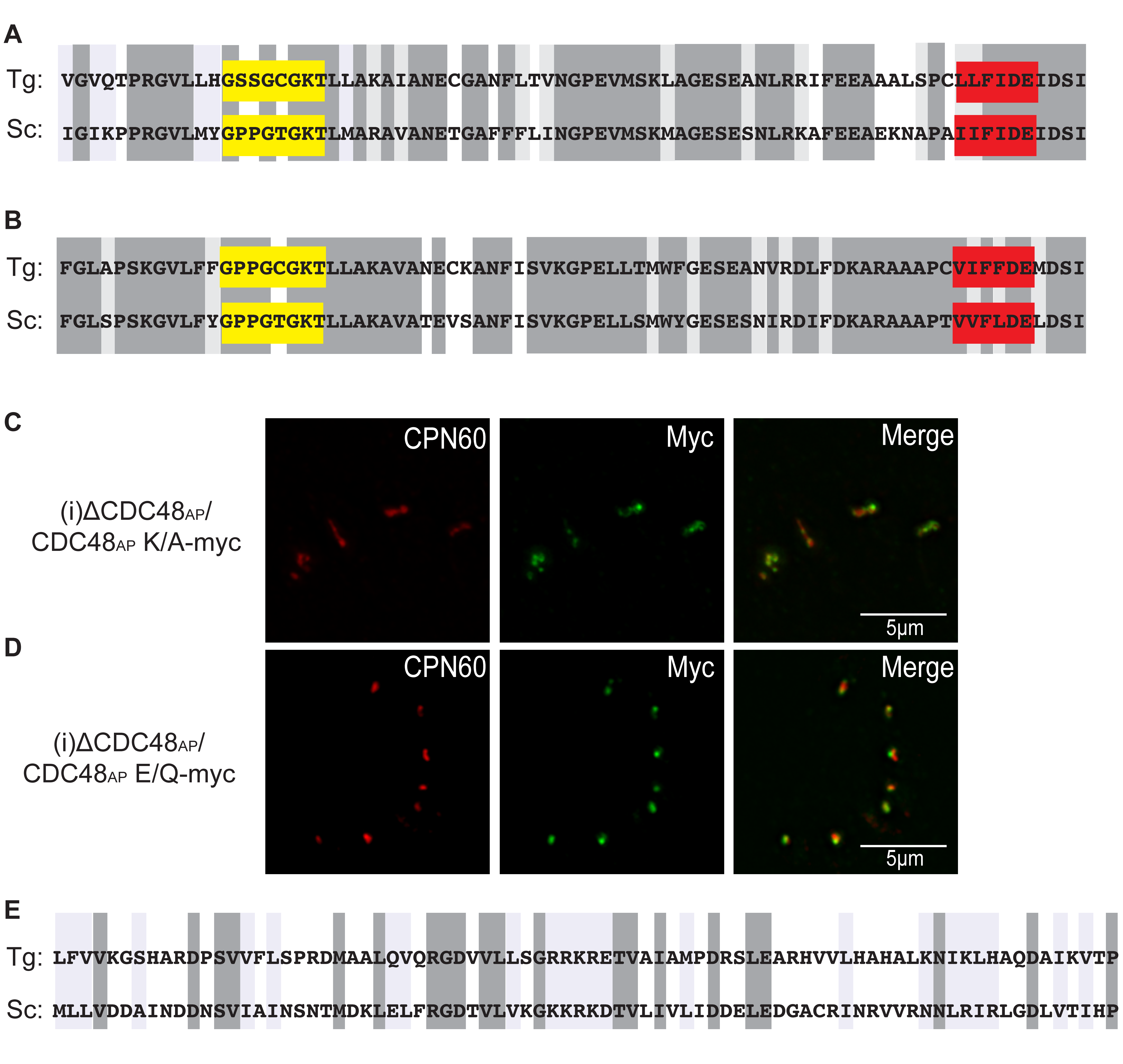

Supplement: FIG S2 [file mbo003173369sf2.tif]

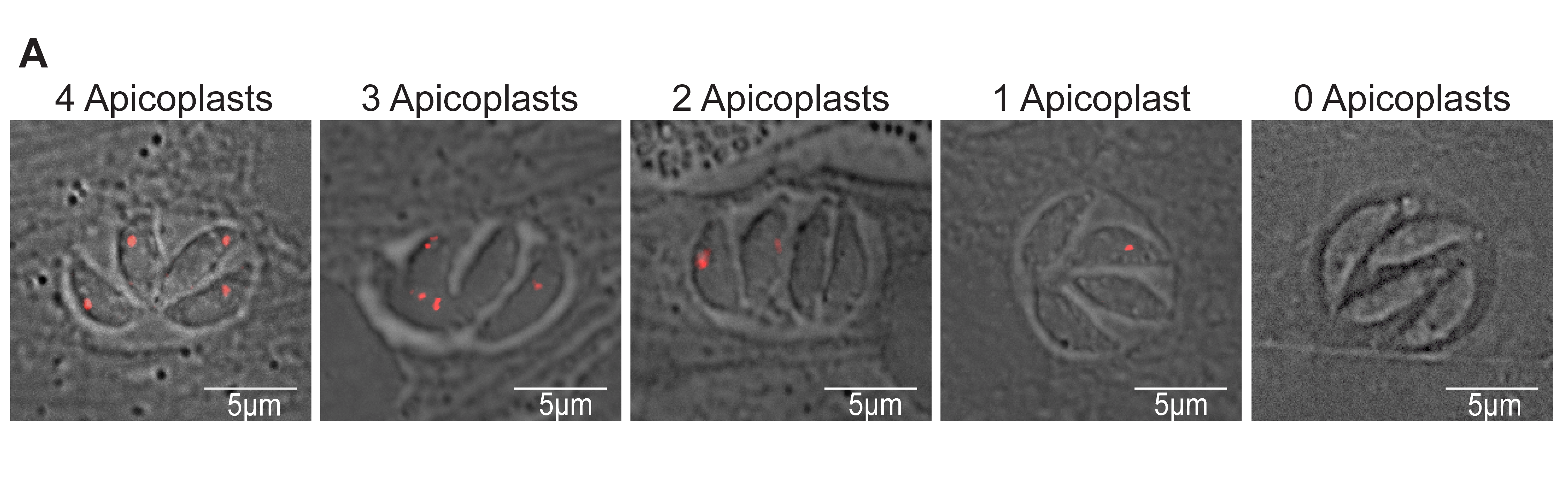

Supplement: FIG S3 [file mbo003173369sf3.tif]

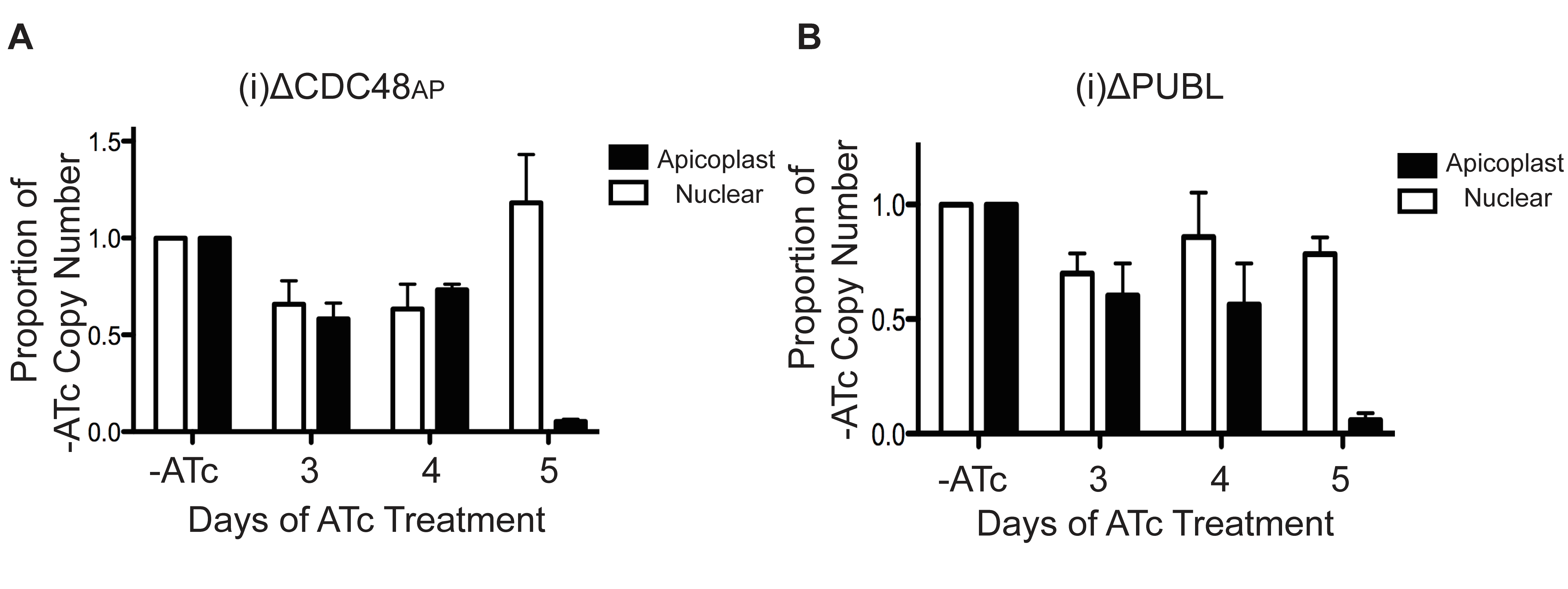

Supplement: FIG S4 [file mbo003173369sf4.tif]

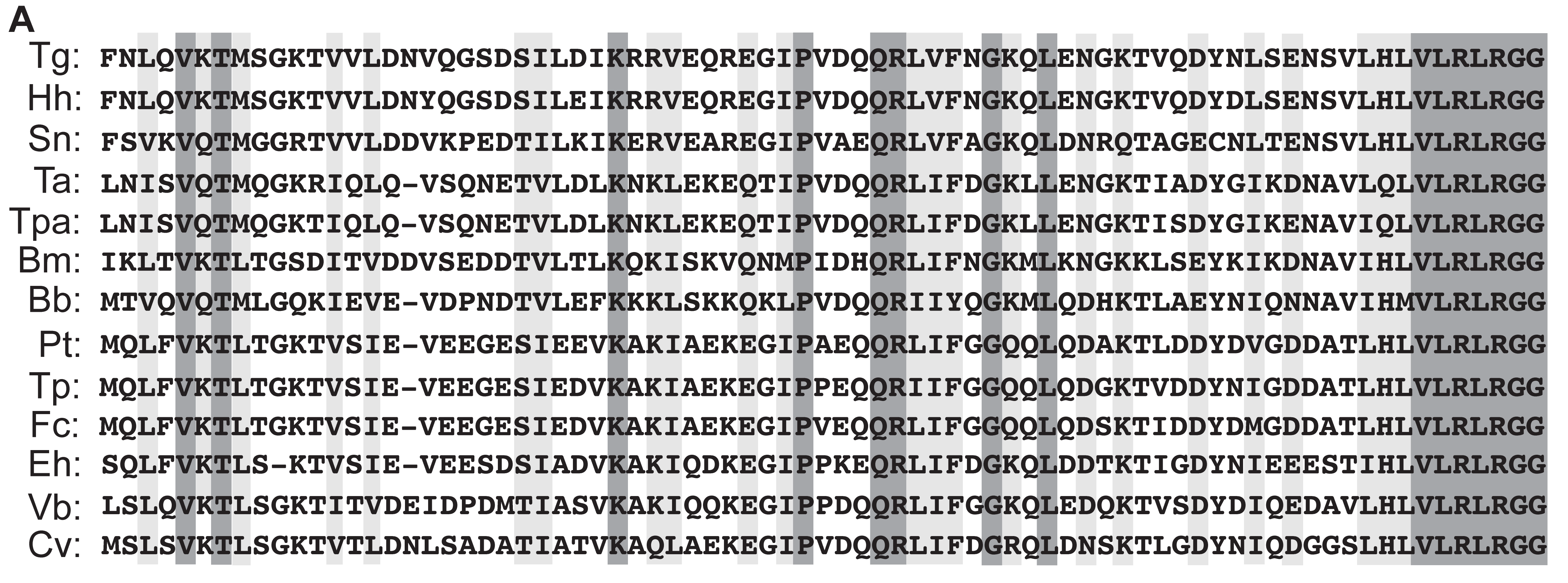

Supplement: FIG S5 [file mbo003173369sf5.tif]

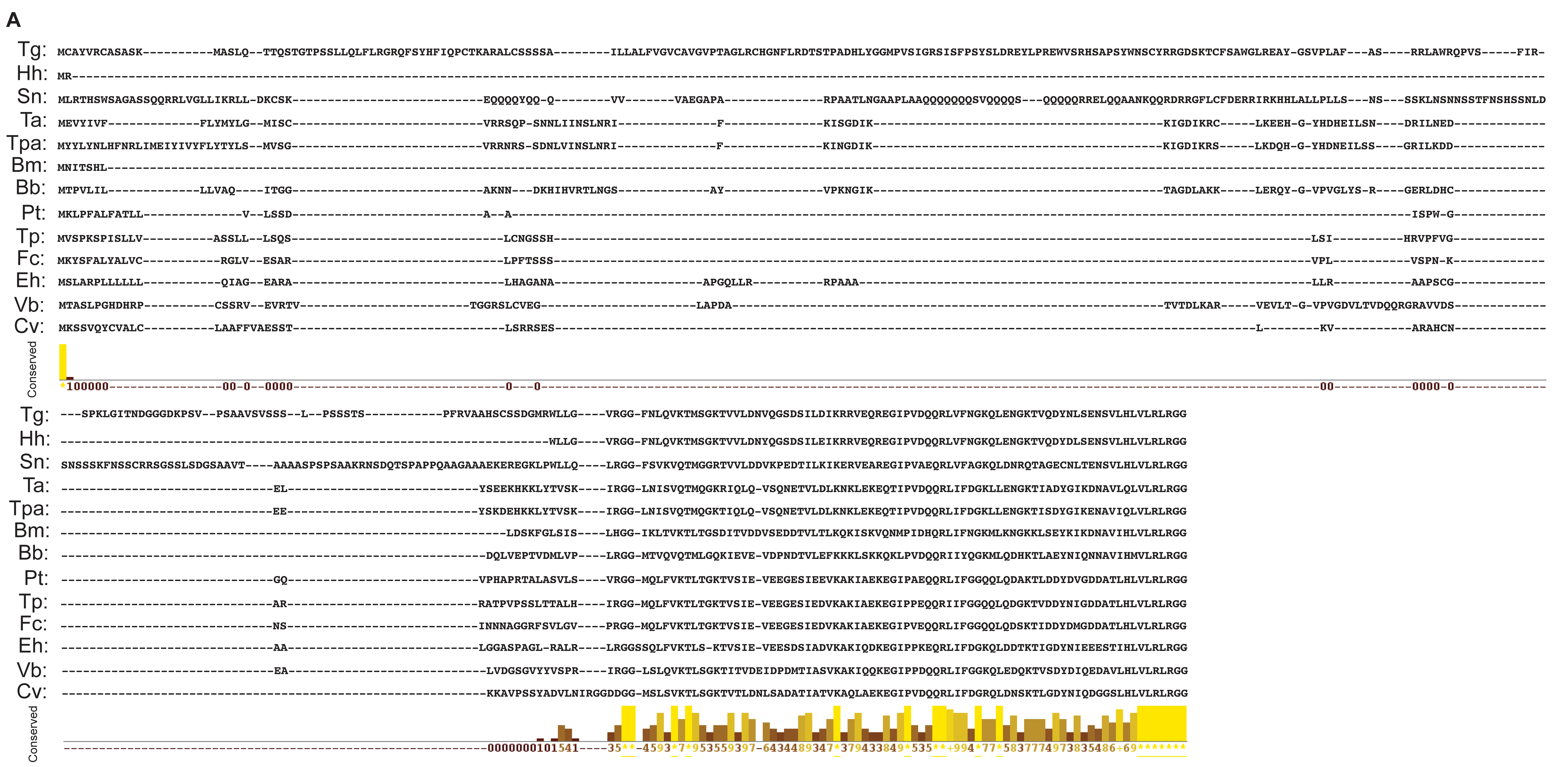

Supplement: FIG S6 [file mbo003173369sf6.tif]

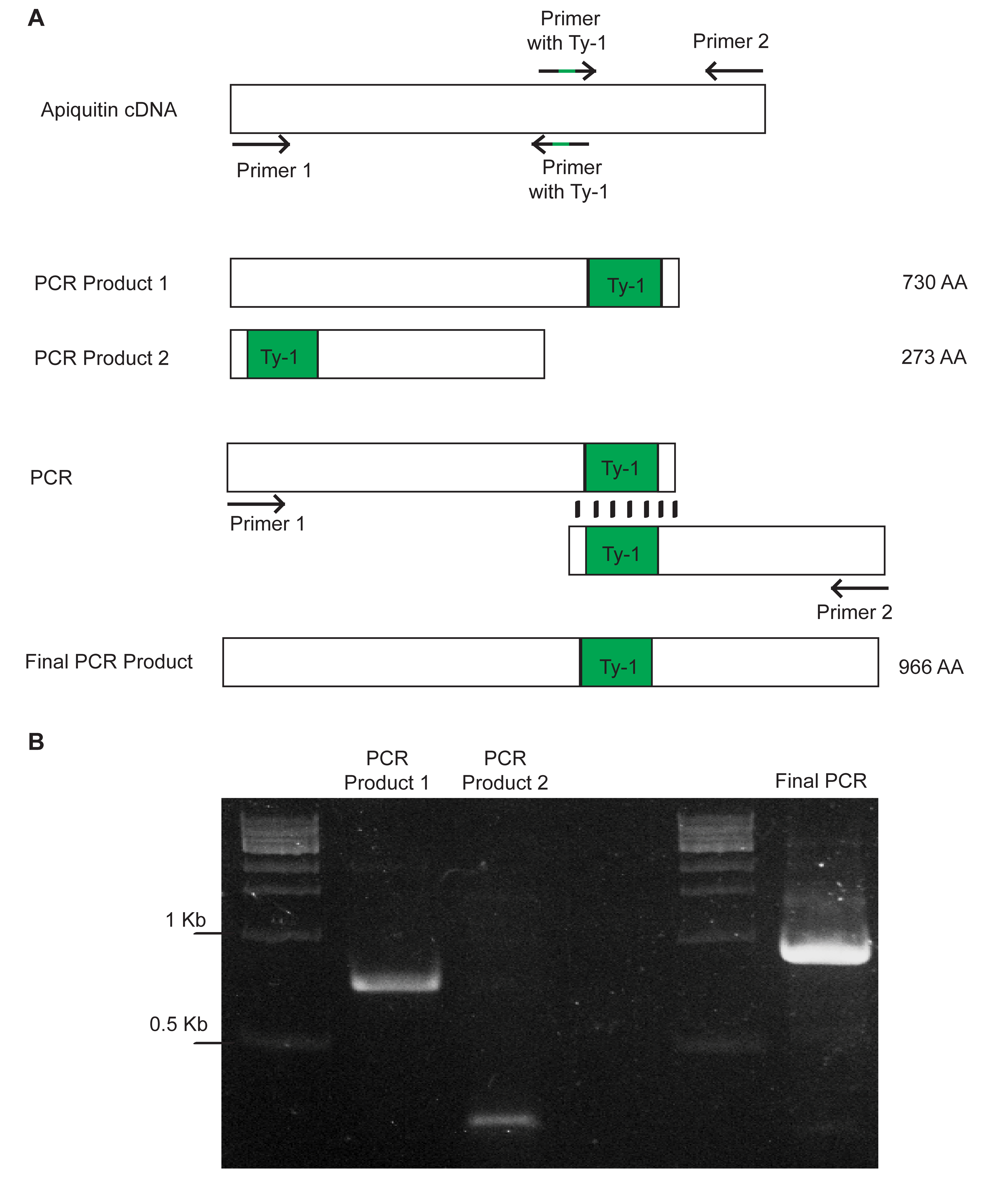

Supplement: FIG S7 [file mbo003173369sf7.tif]

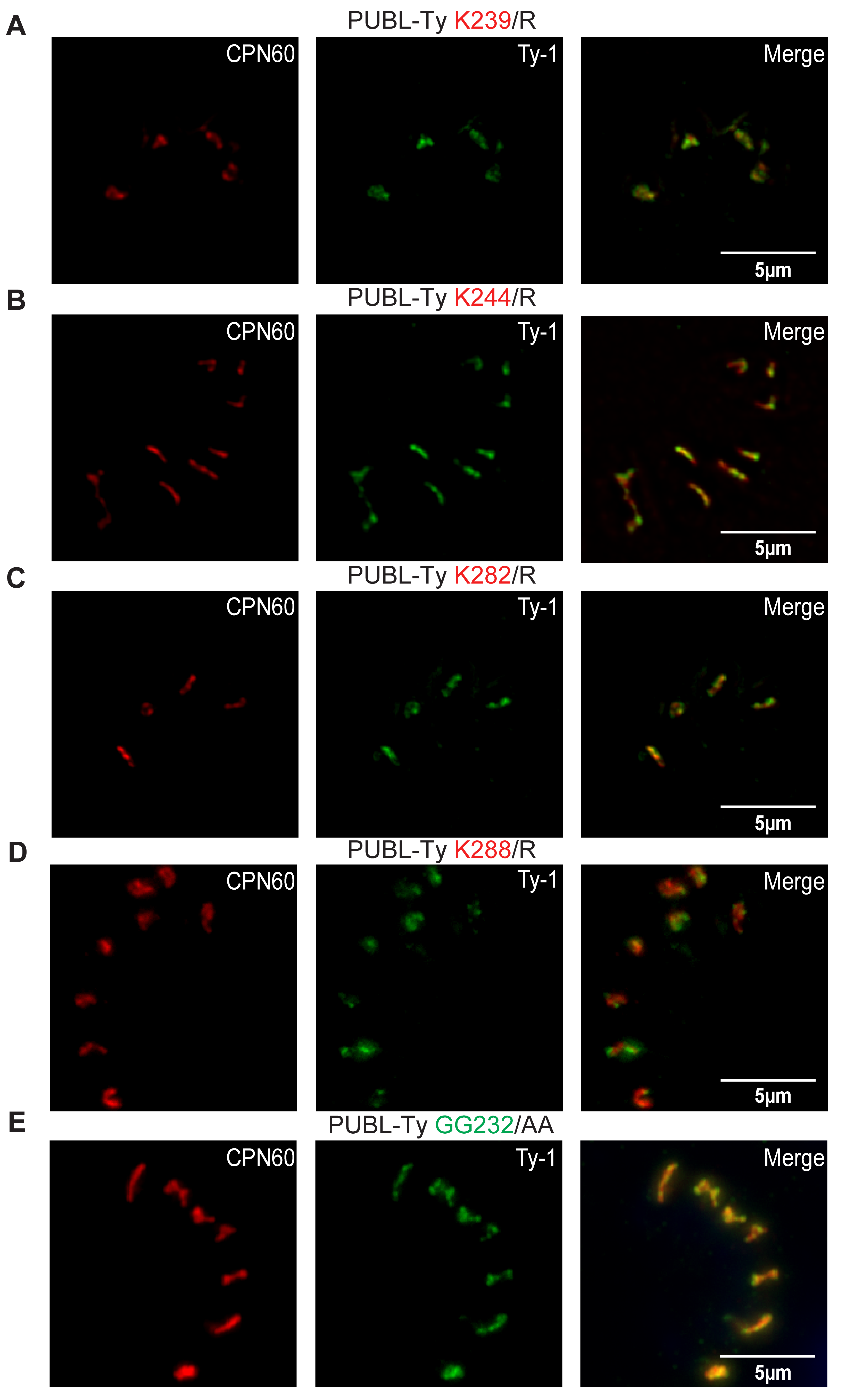

Supplement: FIG S8 [file mbo003173369sf8.tif]
