# Supplementary material for: A Plastid Protein That Evolved from Ubiquitin and Is Required for Apicoplast Protein Import in Toxoplasma gondii
Source: mBio. 2017 Jun 27;8(3):e00950-17. doi: 10.1128/mBio.00950-17 (PMC5487736; doi:10.1128/mBio.00950-17)
Supplement: TABLE S1 [file mbo003173369st1.docx]

**Supplementary Table 1. Primer List:**

| Gene Used | Purpose | Name | Primer Sequence |
| --- | --- | --- | --- |
| PUBL | PUBL-Ty pDC | PUBLEcoRVR | agtgatatcctatctagaaccaccacca |
| PUBL | PUBL-Ty pDC | PUBL5TYR | ctgcatatctagtggatcttgattagtatgaacttcgcctccgccgacgccaagga |
| PUBL | PUBL-Ty pDC | PUBL3TYF | ggaggcgaagttcatactaatcaagatccactagatatgcagatctttgtgaagactcttacc |
| PUBL/Ubiquitin | PUBL/Ub-Ty Chimera pDC | UB5TYR | gttgaaatctagtggatcttgattagtatgaacttcgcctccgcggacgccaagga |
| PUBL/Ubiquitin | PUBL/Ub-Ty Chimera pDC | UB3TYF | ggaaggcgaagttcatactaatcaagatccactagatttcaacttgcaagtcaagacaatgagc |
| PUBLutin/Ubiquitin | PUBL/Ub-Ty Chimera pDC | UB5BglIIF | gatagatctatgtgcgcgtatgtgaggtgtg |
| PUBL/Ubiquitin | PUBL/Ub-Ty Chimera pDC | UB3EcoRVR | agtgatatctcatgcgcctcctcggagt |
| PUBL | PUBL180AApTG | 223125AvrII | agtcctaggtgcgcctcctcggagtctga |
| PUBL | PUBL-Ty 239K/R | PUBL6KRF | gtcttcccgctcattgtcctgacttgcaagttgaaat |
| PUBL | PUBL-Ty 239K/R | PUBL6KRR | atttcaacttgcaagtcaggacaatgagcgggaagac |
| PUBL | PUBL-Ty 282K/R | PUBLKtoR48F | accaacagcgtcttgtattcaacggacgacaactggagaatgg |
| PUBL | PUBL-Ty 282K/R | PUBLKtoR48R | ccattctccagttgtcgtccgttgaatacaagacgctgttggt |
| PUBL | PUBL-Ty 288K/R | UBKtoR54F | cggaaaacaactggagaatgggcgaacagtccaagattacaatctc |
| PUBL | PUBL-Ty 288K/R | UBKtoR54R | gagattgtaatcttggactgttcgcccattctccagttgttttccg |
| PUBL | PUBL-Ty 244K/R | PUBLK11toRF | gtcaagcacgacagtcctcccgctcattgtctt |
| PUBL | PUBL-Ty 244K/R | PUBLK11toRR | aagacaatgagcgggaggactgtcgtgcttgac |
| PUBL | PUBL-Ty 232GG/AA | UBinternalggFty | gattagtatgaacttcgactgcgcggacgccaaggag |
| PUBL | PUBL-Ty 232GG/AA | UBinternalggRty | ctccttggcgtccgcgcagtcgaagttcatactaatc |
| PUBL | PUBL-Ty 309GG/AA | PUBLGGAAF | tgtgatatctcatgcggctgctcggagtctgaggac |
| PUBL | PUBL-Ty 309GG/AA | PUBLGGAAR | tcctcagactccgagcagccgcatgagatatcaca |
| PUBL | PUBL pAVA | UBLicF | ccagggtcctggttcgttcaacttgcaagtcaagac |
| PUBL | PUBL PAVA | UBLicR | cttgttcgtgctgtttatcatgcgcctcctcgga |
| PUBL | (i)ΔPUBL | 223125PRF | acgactaccaaaattaatgcctggaagagaaccgaagctacagagaaaaggaatggtaaccgacaaacgcgttc |
| PUBL | (i)ΔPUBL | 223125PRR | gcaacttgacgggggattaatgcatttcactcttgcagatagtttttcatagatctggttgaagacagacgaaag |
| PUBL | (i)ΔPUBL Promoter | UBPRcheckF | ccatccactatgttgccttc |
| PUBL | (i)ΔPUBL Promoter | UBPRcheckR | tcctttgtggagaaccgct |
| CDC48_AP_ | (i)ΔCDC48_AP_ | CDC48PRF | tctctattatcacagcacacggtcgtcaggagcttttcgacagtacgtgcgaatggtaaccgacaaacgcgttc |
| CDC48_AP_ | (i)ΔCDC48_AP_ | CDC48PRR | gctgaggagtcccagcccgcaccctcgagagggcaccacgcagtccccatagatctggttgaagacagacgaaagc |
| CDC48_AP_ | (i)ΔCDC48_AP_ Promoter | CDC48PRcheckF | gtacacggcgttaatcctg |
| CDC48_AP_ | (i)ΔCDC48_AP_ Promoter | CDC48PRcheckR | tccctctctgagagcgaa |
| T7s4 | T7s4 Promoter | DHFRseqF | cgtttcctcttccctcaaa |
| T7s4/CDC48_AP_ | T7s4 Promoter for (i)ΔCDC48_AP_ | CDC48PRR | gctgaggagtcccagcccgcaccctcgagagggcaccacgcagtccccatagatctggttgaagacagacgaaagc |
| T7s4/ PUBL | T7s4 Promoter for (i)ΔPUBL | 223125PRR | gcaacttgacgggggattaatgcatttcactcttgcagatagtttttcatagatctggttgaagacagacgaaag |
| DHFR | pDC | DHFRSpeIF | agtactagtccaggctgtaaatcccgt |
| DHFR | pDC | DHFRBglIIR | gatagatctcttcccagacacgacaac |
| CDC48_AP_ | ΔCDC48_AP_ K/A-myc | CDC48KAF | cgagcggctgtggagcgaccttgctggcga |
| CDC48_AP_ | ΔCDC48_AP_ K/A-myc | CDC48KAR | tcgccagcaaggtcgctccacagccgctcg |
| CDC48_AP_ | ΔCDC48_AP_ E/Q-myc | Cdc48E829Q rev | ttcgcgatcgagtccatctggtcgaagaagatcacgc |
| CDC48_AP_ | ΔCDC48_AP_ E/Q-myc | Cdc48E829Q sense | gcgtgatcttcttcgaccagatggactcgatcgcgaa |
